# Time Spent Thinking in Online Chess Reflects the Value of Computation

**DOI:** 10.1111/cogs.70119

**Published:** 2025-10-25

**Authors:** Evan M. Russek, Daniel Acosta‐Kane, Bas van Opheusden, Marcelo G. Mattar, Thomas L. Griffiths

**Affiliations:** ^1^ Departments of Computer Science and Psychology Princeton University; ^2^ Department of Cognitive Science University of California San Diego; ^3^ Department of Psychology New York University

**Keywords:** Chess, Decision making, Meta‐reasoning, Planning, Reinforcement learning, Resource rationality

## Abstract

Human planning tends to be efficient, focusing on a relatively small number of options when considering future paths. Recent proposals have suggested that this efficiency reflects intelligent deployment of the limited resources available for planning. A prediction of this and related proposals is that *when* individuals spend time thinking should depend on the benefits and costs of additional computation. We tested this hypothesis by measuring how much time humans spent thinking before acting in over 12 million online chess games. Players spent more time thinking in board positions where additional computation was more beneficial. This relationship was greater in stronger players, and was strengthened by considering only the information available to the player at the time of choice. A simple model based on measuring the actual cost of spending time thinking in online chess was able to capture qualitative features of this relationship. These results provide evidence that the amount of time humans spend thinking is appropriately sensitive to the value of computation.

## 1 Introduction

A central feature of human cognition is the ability to make good decisions in complex environments, despite having limited resources to perform computations (Gershman, Horvitz, & Tenenbaum, 2015; Lieder & Griffiths, 2020). This feature is particularly evident in tasks requiring complex, multistep planning. Identifying the optimal action in such tasks often requires searching through an exponentially large decision tree (Daw & Dayan, 2014). Executing this search demands substantial computational resources, including time and energy. In contrast, the brain has strikingly limited resources, consuming significantly less energy than modern artificial intelligence systems. Such resource limitations are believed to substantially reduce the overall budget for computation. For example, in games like chess and Go, expert humans are believed to search through far fewer future positions than artificial intelligence systems (Charness, 1981; Silver et al., 2018).

How then do sufficiently experienced humans perform well in computationally demanding tasks like complex, multistep planning? Recent proposals in cognitive science have suggested that a key may be an ability to make intelligent *meta*‐decisions about when and how to allocate limited cognitive resources (Boureau, Sokol‐Hessner, & Daw, 2015; Lewis, Howes, & Singh, 2014; Lieder & Griffiths, 2017; Shenhav, Botvinick, & Cohen, 2013). Investing greater resources on computations—such as performing a deeper tree search—can improve decision quality and lead to better outcomes. But, computation is also costly, consuming time and energy. Strategically choosing *when* to compute—only when the expected benefits outweigh the costs—may help explain how people succeed in complex planning despite such limitations.

Do people base decisions about whether to compute on the expected benefits and costs? Research in related areas has typically used simple tasks in controlled lab settings (Frömer et al., 2021; Grahek, Frömer, Prater Fahey, & Shenhav, 2023; Kool, Gershman, & Cushman, 2017; Lieder & Griffiths, 2020; Otto & Daw, 2019; Otto, Braem, Silvetti, & Vassena, 2022). This has resulted in several limitations, particularly in generalizing findings to complex planning. First, in simple tasks, the benefits of computation are manipulated by altering outcome payoffs across a small number of discrete conditions. In more complex tasks, however, the benefits of computation more often vary with potential improvements in decision quality. As a result, sensitivity to these more nuanced benefits is typically not measured. Second, testing cost−benefit trade‐offs requires a specification of the cost of computation. However, in most existing work, this cost is not measured but assumed. A final limitation concerns the generalization of these findings beyond controlled lab tasks. Studying behavior in less controlled contexts, such as games, may provide insights into how these cognitive processes operate in more general situations (Allen et al., 2024; van Opheusden & Ma, 2019; Wise, Emery, & Radulescu, 2024).

The ability to make effective decisions under resource constraints is likely a key component of developing expertise within a domain. However, it remains unclear whether expertise development involves learning to allocate limited cognitive resources more effectively. One reason for this gap is that most prior work on resource allocation has used standard laboratory tasks, which are poorly suited for studying expertise. Participants typically do not engage with these tasks enough times to develop true expertise. In contrast, more naturalistic domains—such as chess—have long served as testbeds for studying expertise. This research has primarily examined how cognitive representations (e.g., memory structures; Chase & Simon, 1973; Gobet & Simon, 1998; 2000) and decision processes (e.g., search depth; Charness, 1981; van Opheusden et al., 2023) change with experience. However, how expertise affects the intelligent allocation of limited cognitive resources remains largely unexplored.

**Fig. 1:**
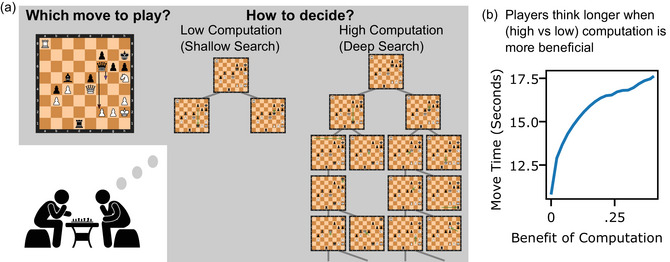
Approach and preview of results. (a) Recent proposals have suggested that humans succeed in complex tasks by making intelligent decisions about when to spend limited cognitive resources on computations. In chess, this entails intelligently deciding not only which move to play (left), but also how much resources should be spent performing computations to make this decision. Specifically, players should spend more resources deciding, when the benefit of performing computation is more beneficial. We operationalize this benefit of additional computation by measuring how move quality changes when deciding using a (high computation) deep tree search compared to a (low computation) shallow tree search (right). (b) To preview our results, we find that players’ move times are sensitive to this benefit of computation. The plot shows players’ mean move times as a function of the benefit of computation from 600+0 time‐control setting (defined below).

Here, we test whether people make intelligent decisions about allocating limited cognitive resources in complex planning tasks, and how this ability varies with domain‐specific expertise. We do so by analyzing over 12 million online chess games. In this naturalistic setting, we use time spent per move as a measure of cognitive resource allocation. To evaluate whether players are sensitive to the benefits of applying computation, we estimate the relative value of searching deeper down a decision tree from each position (see Fig. 1a for an overview). Leveraging this dataset, we precisely measure how average planning time varies with the estimated benefit of computation (see Fig. 1b for a preview of the key result).

To outline the rest of this article, we begin with an overview of frameworks proposing that humans make intelligent decisions about when to allocate limited cognitive resources. We then examine the extent to which these frameworks’ predictions have been tested in lab‐based tasks, followed by prior research on time allocation and expertise development in chess. Next, we introduce our approach to testing the prediction that humans strategically allocate cognitive resources in online chess, leveraging an artificial‐intelligence‐based chess engine and a large naturalistic dataset. Finally, we test key predictions of this framework: (1) move times reflect the benefit of thinking and are linked to expertise; (2) players incorporate uncertainty in estimating these benefits; and (3) move times account for the cost of spending time. Altogether, this demonstrates that human cognition involves intelligently considering the benefits and costs of computation.

## 2 Background

### 2.1 Rational metareasoning and related frameworks

Rational meta‐reasoning was originally developed in artificial intelligence research to provide a framework for how systems should allocate limited computational resources (Russell & Wefald, 1991). Recent proposals in cognitive science have suggested applying this framework to explain how people manage to make good decisions in spite of their limited cognitive resources (Lieder & Griffiths, 2017). Rational meta‐reasoning treats the act of performing a computation itself as a decision—one that should be evaluated through a cost−benefit analysis. Computations are beneficial when they improve estimates of which option is best, leading to better choices. But, when resources are limited, computations are costly to the extent that they leave fewer resources available for future situations. Computations should be executed only when the expected benefits of execution exceed the costs.

While rational meta‐reasoning was developed in the AI literature, analogous frameworks have been developed in cognitive neuroscience, psychology, and economics. For example, in cognitive neuroscience, several models have been developed that treat mental effort, self‐control, and model‐based planning as the result of cost−benefit decisions about how to spend limited cognitive resources (Boureau et al., 2015; Keramati, Dezfouli, & Piray, 2011; Kool et al., 2017; Kool & Botvinick, 2018; Shenhav et al., 2013, 2017). These accounts build on earlier work in cognitive science examining how mental effort is defined and how it is traded off against potential rewards (Gigerenzer & Todd, 1999; Payne, Bettman, & Johnson, 1993; Thomson & Oppenheimer, 2022). Across these accounts, a shared prediction is that mental processes balance the benefits and costs of computation.

Importantly, although these frameworks use a decision‐theoretic approach to model how people allocate limited cognitive resources, they do not assume that people explicitly weigh these costs and benefits. Instead, they suggest that behavior follows a policy that approximates the results of such a cost−benefit comparison. The prediction is made at Marr's computational level of analysis, which defines the abstract objectives of a system and the ideal way to satisfy them (Marr, 1982). At the algorithmic level, different strategies or heuristics could implement this policy in practice—yielding behavior that is consistent with efficient resource use.

### 2.2 Sensitivity of mental effort to benefits and costs of computation

Many studies have shown that people adjust how much cognitive control and mental effort they deploy, based on expected rewards, costs, and effectiveness (Frömer et al., 2021; Grahek et al., 2023; Hall‐McMaster, Muhle‐Karbe, Myers, & Stokes, 2019; Hübner & Schlösser, 2010; Otto et al., 2022; Otto & Vassena, 2021). Similar approaches have also been used to model decision times in sensory discrimination tasks (Drugowitsch, Moreno‐Bote, Churchland, Shadlen, & Pouget, 2012) and value‐based decision‐making (Lee & Daunizeau, 2021; Tajima, Drugowitsch, & Pouget, 2016). These studies suggest that both humans and animals adjust how long they deliberate, balancing potential improvement in decision accuracy against the cost of time—though some work challenges this view (Bhui, 2019; Oud et al., 2016).

A number of theoretical models propose that decisions about *when* to plan should follow a similar principle of cost−benefit comparison (Agrawal, Mattar, Cohen, & Daw, 2022; Keramati et al., 2011; Mastrogiuseppe & Moreno‐Bote, 2022; Mattar & Daw, 2018; Sezener, Dezfouli, & Keramati, 2019). These models treat planning computations—such as additional steps of tree search—as actions that improve the agent's estimate of an option's utility, leading to better choices. Some of these models make a simplifying assumption that the agent has “oracle” knowledge that exactly prescribes how a planning computation will change an action's estimated value (Agrawal et al., 2022; Mattar & Daw, 2018). Other models treat the agent as holding probabilistic beliefs about the values of different actions (Keramati et al., 2011; Sezener et al., 2019). These beliefs also prescribe uncertainty about how values may change as a result of applying planning computations. Planning computations reduce uncertainty in these beliefs, either partially or fully. The agent must weigh the benefit of this uncertainty reduction against the cost of computation when deciding whether— and how much— to plan.

Despite the influence of these models, they have mostly been tested in simple reinforcement learning tasks (Bolenz, Kool, Reiter, & Eppinger, 2019; Kool et al., 2017). These tasks are far less complex than real‐world planning (van Opheusden & Ma, 2019), and their relationship to planning is debated (Akam, Costa, & Dayan, 2015; Wang et al., 2018). As a result, it remains unclear whether these paradigms explain how people allocate planning computations in more complex tasks.

Although there are likely many reasons why computations are costly, recent work in cognitive control and planning has focused on the opportunity cost incurred from the time they consume. In tasks where agents aim to maximize average reward rate, the time spent on any action carries an opportunity cost that increases linearly with the average reward rate (Bertsekas, 2012). As a result, the average reward rate has been widely used as a proxy for the cost of time in theories of foraging (Stephens & Krebs, 1986), vigor (Niv, Daw, Joel, & Dayan, 2007), sensory decision‐making (Gold & Shadlen, 2002), strategy selection (Lieder & Griffiths, 2017), control allocation (Otto & Daw, 2019), planning (Agrawal et al., 2022; Keramati et al., 2011; Sezener et al., 2019), and social media engagement (Lindström et al., 2021). However, when the goal is not to maximize reward rate, the average reward rate is no longer a valid stand‐in for the cost of time. We explore this issue in chess, where the objective is not to maximize reward rate, but rather to achieve checkmate before clock time runs out.

### 2.3 Use of time and expertise in chess

Our investigation of whether move times are sensitive to the benefits and costs of computation dovetails with research in chess itself. The problem of deciding how long to search has been considered in the computer science of developing engines for chess and other games (Baier & Winands, 2016; Beal, 1990; Burduli & Wu, 2023; Donninger, 1994; Hay et al., 2012; Huang, Coulom, & Lin, 2010; Hyatt, 1984; Kocsis, Uiterwijk, & van den Herik, 2001; Markovitch & Sella, 1996; Newell & Simon, 1972; Turing, 1953; Vuckovic & Solak, 2009), leading to several heuristic strategies. For example, this decision may be based on the stage of the game, the complexity of the current position, or it may be dynamically adjusted as the engine searches the tree. Additionally, time management is a core skill in competitive chess, and players train their intuition for identifying critical positions in which it is worth spending time (Naroditsky, 2019; Rosen, 2017). Our contribution here is to study the problem empirically, developing an approach to quantify intelligent use of time and to using a large‐scale dataset to investigate whether players show signatures of such intelligence.

Chess and other games have also been used for the study of expertise. A prominent early theory proposed that expertise depends on the storage of “chunks”—groups of frequently co‐occurring pieces, along with typical moves and valuations—in long‐term memory (Chase & Simon, 1973). Later refinements to this theory proposed a more complex retrieval structure, templates, and larger sets of pieces that experts could amend to fit specific positions (Gobet & Simon, 1996). This proposal has explained differences in retrieval between amateur and expert players (Gobet & Simon, 1998), and also in how retrieval changes as presentation time varies (Gobet & Simon, 2000). An alternative perspective has highlighted improvements to decision‐tree search. Think‐aloud paradigms (Charness, 1981), and modeling of human choices in simpler two‐player games, suggest that experts generally search deeper than nonexpert players (van Opheusden et al., 2023). However, some have argued that this deeper search itself may be facilitated by larger chunks or templates (Gobet, 1997).

Prior work has also examined how move quality changes with additional time. These studies show that both experts and novices tend to make better moves when given more time (Burns, 2004; Chabris & Hearst, 2003; Klein et al., 1995; Medvegy et al., 2022; Moxley et al., 2012). Here, we test whether an additional component of expertise is sensitivity to the benefits and costs of computation when selecting how much time to plan. Is intelligent deployment of limited computational resources a component of achieving expertise in a cognitive domain?

## 3 Measuring intelligent use of limited resources with AI and a massive naturalistic dataset

Our approach to studying the factors influencing chess players’ move times makes use of two novel resources. The first is a massive online dataset of online chess games, which contains information about the time players spend in different positions. The second is an open‐source AI‐based chess engine, which we utilize to quantify measurements of the benefits and costs of computation.

### 3.1 Stockfish chess engine

In our main analysis, we use the Stockfish chess engine to measure the benefits and costs of computation. Stockfish, like other AI‐based chess engines (Campbell, Hoane, & Hsu, 2002; Silver et al., 2018), and models of human decision‐making in games (van Opheusden et al., 2023), employs heuristic search to estimate a player's advantage from different board positions. Heuristic search involves two components: a static evaluation function that provides a quick estimate of a player's advantage and a search process that refines this estimate by building a decision tree.

The decision tree expands by evaluating potential moves for both players up to a certain depth (*N* moves), applying the static evaluation function to each leaf node (end position), and computing the maximal leaf value obtainable under opponent actions taken to minimize this value. Stockfish specifically uses Principal Variation Search, expanding the search tree one depth at a time while determining the best move sequence and position value at each depth. It also applies various heuristics to optimize the search order and prune the decision tree efficiently. For heuristic evaluation, Stockfish relies on a neural network trained through supervised learning.

We use Stockfish to measure the effects of computation—specifically, how move preferences shift with additional computational resources—and as an approximation of the true evaluation and optimal move in each position. Our use of Stockfish is a practical choice, aiming to capture the effects of computation at a high level by comparing results from different search depths (e.g., depth 1 vs. depth 15). Two aspects of our approach to using Stockfish make this approach appropriate for our analysis. The first is that we examine the effects of increased computation at a coarse level (primarily comparing effects depth 1 vs. depth 15 searches on move selections), thus abstracting away from particulars of how node‐to‐node choices about where to search are made, which would vary between different approaches. The second is that we aggregate over massive amounts of data, and thus can capture effects of computation in aggregate, rather than for any particular position. Although Stockfish may not perfectly model human search behavior in chess, heuristic search algorithms like Stockfish have been shown to effectively model human behavior in two‐player games (van Opheusden et al., 2023). Moreover, Stockfish's prediction of human moves (35−40% of the times; McIlroy‐Young, Sen, Kleinberg, & Anderson, 2020) compares favorably to this recent cognitive model (Kuperwajs et al., 2023; van Opheusden et al., 2023).

In the Supplementary Materials (Section *Interrogating the validity and necessity of assumptions underlying the benefit of computation)*, we validate key assumptions underlying our use of Stockfish to measure the effects of computation (Figs. S2 and S3). We also test the robustness of our findings against a variety of methods for computing position value and the effects of computation (Figs. S4–S8), which alter these assumptions. These results indicate that our findings are not specific to the use of the Stockfish algorithm to measure the benefit of computation, but are applicable to the use of heuristic search approaches more broadly.

### 3.2 Lichess.org data

We use a dataset of chess games from the popular online chess server Lichess.org, which contains move and timing information from all games played on the site. For games played on the site, both players have a clock that counts down as they spend time on their move. Following each move, some number of seconds, referred to as an increment, can be added back to the player's clock. Players lose when they run out of clock time. Games are played across a variety of time‐control settings, which specify the amount of time that players start with on their clock, as well as the increment. To denote a time‐control setting, we use the notation *S*+*I*, which indicates that each player starts with *S* s on their clock and gets back *I* s following each move as an increment. Relevant for our analysis of expertise, the website maintains an Elo rating for each player in each grouped collection of time‐control settings (e.g., “Bullet” includes 60+0 and 120+1, “Blitz” includes 180+0, 180+2, 300+0, 300+3, etc.). Elo specifies an estimate of a player's overall playing strength, relative to everyone else on the site, within those time‐control settings. Higher Elo reflects a stronger player. A player's Elo rating is updated each game depending on the outcome and the rating difference between a player and their opponent. A player whose rating is 100 points greater than their opponent would be expected to win 64% of the time (Elo, 1978).

### 3.3 Approach

We use these new resources to study whether players’ decisions about when to spend time thinking reflect an intelligent balance of the associated benefits and costs. Our specific approach is to develop a measure that can be approximated with chess engines, the *benefit of computation*, which estimates the net utility of spending computational resources identifying the optimal move compared to selecting a move that could be identified with fewer resources. Given a measurement of the benefit of computation, we first examine the relationship between this benefit and the time players spend thinking, as well as how this relationship varies with expertise. Next, we refine the measure of the benefit of computation to rely only on the information available to players at the time of their decision, and we assess whether this adjustment explains additional variance. Finally, we evaluate whether players’ move times reflect sensitivity to the actual costs of time expenditure in online chess games. We analyze the predicted relationship between move times, the benefit of computation, and time‐control settings based on the measured cost‐structure, and then determine if this pattern is observed in players’ actual move times.

## 4 General methods

### 4.1 Data

**Fig. 2:**
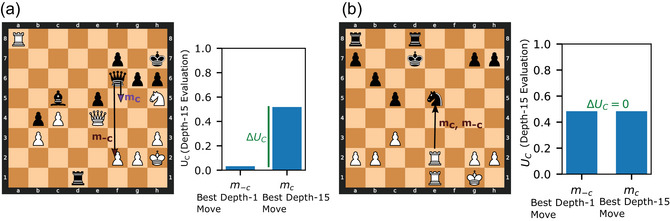
Benefit of computation. $\Delta U_{C}$ is defined as the difference in true board position advantage, $U_{C,}$ resulting from performing the optimal move, $m_{C}$, which would be selected if computation were performed, and the move which would be selected if computation were not performed, $m_{-C}$. Operationally, $m_{-C}$ is identified through depth‐1 Stockfish search, and $U_{C}$ is defined as depth‐15 Stockfish evaluation, and $m_{C}$ is the move that maximizes $U_{C}$. (a) Example position with high benefit of computation. Left: At depth‐1, Stockfish recommends using the queen to capture a pawn, $m_{-C}$= Qxf2. However, a deeper search reveals that moving the queen away from the black king allows white to launch a winning attack, starting with a rook sacrifice and ultimately achieve a checkmate through a 5‐move sequence involving the queen and knight. This attack can be prevented with $m_{C}$ = Qf5, offering a trade of queens. Right: The benefit of computation in this position is thus high as it leads to the selection of a move that prevents a loss. (b) Example position with low benefit of computation. Left: Rxe5 maximizes both Stockfish's depth‐1 and its depth‐15 value function, and thus $m_{C}$ and $m_{-C}$ are the same. Right: Because computation does not cause the selected move to change, it provides no benefit in this position.

**Fig. 3:**
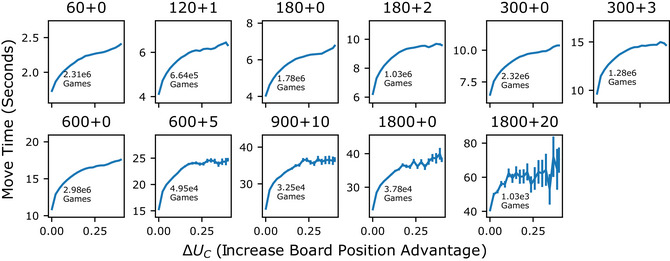
Time spent thinking relates to the benefit of computation. Each panel shows a different time‐control setting, where the first number indicates the number of seconds a player's clock starts with, and the number following the + indicates the increment following each move. Move times were positively related to the ∆$U_{C}$ in every time‐control setting. This relationship was concave and increased with increasing total time and increment.

**Fig. 4:**
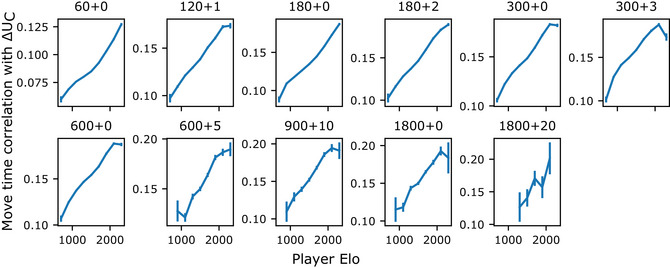
Effect of playing strength on the relationship between move time and benefit of computation. Each plot shows Spearman correlation (computed separately for each player in each game) between move times and benefit of computation, ∆$U_{C}$, for players binned by Elo, for a given time‐control setting. We include only bins with at least 200 games.

**Fig. 5:**
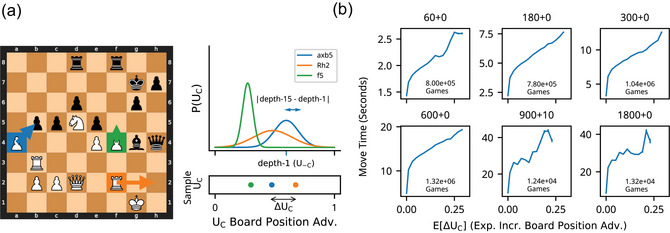
Expected benefit of computation. (a) Example position. Left: Arrows indicate three moves under consideration. Top Right: For each move in consideration, a probabilistic estimate of the result of computation is determined, $P(U_{C}(m)).$ The distribution is a Gaussian with a mean at the depth‐1 evaluation, $\mu =U_{-C}\left(m\right),$ and standard deviation corresponding to the absolute difference between a depth‐1 and depth‐15 evaluation, $\sigma =|U_{C}\left(m\right)\;-\;U_{-C}\;\left(m\right)|.$ Bottom Right: An illustrative sample of $U_{C}(m$) from the distribution of $P(U_{c}\left(m\right))$ for each move. In this sample, Rh2 is now the best move and $\Delta U_{C}$ would be the difference in utility between this new best move (Rh2) and the move which would be selected without computation (axb5). The full expected benefit of computation, $E[\Delta U_{C}]$, is computed as an expectation over the distributions, $P(U_{C}\left(m\right))$, of all moves, m, under consideration. (b) Move times were related to the expected benefit of computation, $E[\Delta U_{C}].$ Move times were positively related to the $E[U_{C}]$ in every time‐control setting (separated by panel). This relationship was concave and was larger in settings both with more total time and where players got more time back following each move.

**Fig. 6:**
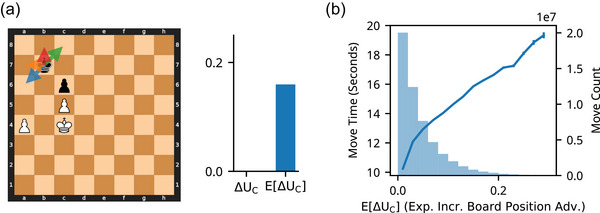
The expected benefit of computation explains additional variance. (a) Example position with a high expected benefit of computation and true benefit of 0. Arrows denote the top four moves under consideration. While depth‐1 Stockfish only weakly prefers Ka6, a deeper search reveals that only this move prevents white's plan of moving the king to d6 and then using the a‐pawn to deflect black from the defense of c6. Since the preferred move with and without computation is the same, the true benefit of computation, $\Delta U_{C}$, is 0. However, the large possible variation of the utility of each move after computation contributes to a high expected benefit of computation. (b) Move times were related to the expected benefit of computation when the true benefit is 0. The histogram shows the distribution of the expected benefit of computation when the true benefit of computation is 0 in the “600+0” time condition. Move times are still positively related to $E[\Delta U_{C}]$ even when the true benefit of computation is 0.

We analyzed a subset of games played on Lichess.org (https://database.lichess.org/) in 2019 that were previously analyzed in McIlroy‐Young et al. (2020) and available at http://csslab.cs.toronto.edu/datasets/#maia_kdd. The analyzed games were played in 2019, were from one of the 11 default time‐control settings, and were sampled in the order that they appear in the data. Data underlying Figs. 2, 3, and 6 consist of 519 million moves from 12.49 million games. Data underlying Figs. 4 and 5 consist of 221 million moves from 5.17 million games (see Tables S1 and S2 for the number per time‐control). The number of games was selected such that analysis would take a month of time running on the university's computer clusters. We limited our analysis to moves between the 15th and 75th ply (a ply is a move by a single player) of each game to minimize the influence of preplanned openings and potentially abnormal play occurring in the endgame of excessively long games.

### 4.2 Stockfish evaluation and move selection

We used Stockfish version 14 for all evaluations, and interfaced with this through the Python chess package. To remove order dependence, we cleared the hash prior to every evaluation. Stockfish's evaluation is returned either in units of centipawns or distance, in number of move‐plys, from mate. To place these separate units on a common scale, we converted each of these units to a new measure of position advantage by fitting two logistic regression models, which mapped either centipawn advantage or distance from mate (for the active player; evaluated at depth‐15) to the probability that the active player won the match. This measure of board position advantage thus corresponded to a time‐agnostic win probability. Moves used to fit the models were filtered such that both players had over 60 s left on their clock and were from games with 300+0 and 600+0 time‐control settings, which ended in checkmate. Note, we only applied this filtering for purposes of defining this mapping from Stockfish evaluation to win probability. Games used in the main analysis were not similarly filtered.

Computation of the benefit of computation requires identifying the best move following a depth‐15 search, $m_{c}$. However, due to the pruning heuristics Stockfish employs, the best move is not always identified. To address this, we defined a consideration set of moves, $m_{i},$ by running a depth‐16 search and recording each move Stockfish selected as its preferred move after completing each iterative depth from 1 to 16. We then computed $U_{C}\left(m_{i}\right)$ for each move in the set and selected $m_{C}$ as the move with the maximum value.

### 4.3 Computing move times

We computed move times as the difference in clock between a player's successive moves, minus the time‐control specified increment. We note that on Lichess, it is possible to add time to an opponent's clock; however, we believe this rarely occurs due to our exclusive analysis of competitive games in which players’ Elo is affected. Additionally, due to the large number of games analyzed, this does not affect our analysis.

### 4.4 Statistics

To obtain estimates and *p*‐values for relationships between either the benefit of computation or the expected benefit of computation and move times, we ran linear regressions predicting move times as a function of ∆$U_{C}$, player Elo, and their interaction. Except when measuring effects of time‐control setting itself, all regressions included separate intercepts and predictor variables for each time‐control setting (statistics in the supplement are thus listed by time‐control setting). For measuring the effects of time‐control setting directly, we do not split regressors by time‐control setting but instead include both increment and total time, as well as their interaction with the benefit of computation, as additional predictor variables. To measure whether the effect of benefit of computation on move times is concave, we repeat these same regression but, substitute $\sqrt{\Delta U_{C}}$ for ∆$U_{C}$, and compare the Akaike information criterion of these models. Additionally, to compare the the influence of benefit of computation and the expected benefit of computation on move times, we similarly compare regression models by Akaike information creterion.

To handle the nonindependence of moves made for a given player, we include the Elo rating as a covariate in all regression analyses, as mixed‐effects regression is not computationally feasible for this amount of data. This approach is supported by the demonstration that accounting for Elo is sufficient to predict a player's moves with high accuracy (McIlroy‐Young et al., 2020). Given the large dataset, this choice does not affect the results. For all regressions, player Elo ratings were centered at 1500. We computed linear regression estimates and *p*‐values using the R package biglm (Lumley, 2011), which allows for incremental computation of linear regressions without needing to hold all data in memory simultaneously. In presenting the main results, we focus primarily on the qualitative effects of the relationship between move times and the benefit of computation, going beyond mere tests of significance.

## 5 Relationship between move times, benefit of computation, and expertise

We first examine whether move times are influenced by the benefit of planning computations, and how this relationship changes with expertise.

### 5.1 The benefit of computation

Here, we describe how we measure the benefit of computation for a given position. Planning computations are beneficial to the extent that they lead a player to make a move that results in a more favorable position. We quantify this reasoning to define the benefit of applying computation in a given board position. Following the standard approach for artificial‐intelligence‐based chess engines (https://stockfishchess.org/; Silver et al., 2018), models of human decision‐making in games (van Opheusden et al., 2021), and models of meta‐reasoning (Russell & Wefald, 1991), we assume that players start each turn with an initial estimate of the utility of candidate moves, $U_{-C}$, which involves no planning. Here, utility provides a measure of the player's board position advantage, not taking into account the time remaining on the clock (*U* = 1 reflects certain victory, *U* = 0 reflects certain loss, see Methods; Takeuchi, Kaneko, Yamaguchi, & Kawai, 2007). Players can then either make the maximum‐utility move $m_{-C}=\mathrm{argma}x_{m}[U_{-C}(m))],$ or perform a planning computation. We assume that planning can provide the true utility of moves, $U_{C}$, and thus allow players to select the optimal move, $m_{C}$ = [($m_{C}=\mathrm{argma}x_{m}[U_{C}(m)],$)]. Although this framing of computation as an all‐or‐nothing choice between planning or no planning is a simplification, it enables us to measure the benefit of computation quantitatively using available tools, and as we will demonstrate, it still confers substantial explanatory power. Under this framing, the benefit of computation is the increase in true board position advantage resulting from the optimal move $m_{C}$ versus the move that could be identified with less computation, $m_{-c}$:

$$\Delta U_{C}=U_{C}\left(m_{C}\right)-\;U_{C}\left(m_{-C}\right).$$

To provide intuition, ∆$U_{C}$ is high when computation changes the preferred move, $m_{C}\;\neq \;m_{-C}$, and the newly preferred move improves the board position relative to the previously preferred move (Fig. 2a). When computation does not change the preferred move, $m_{C}=m_{-C}$, and $\Delta U_{C}=0$ (Fig. 2b).

Given a definition of the benefit of computation, our approach is to measure this in practice using the Stockfish chess engine, and to use the Lichess dataset to examine whether relationships between this and the time players spend thinking prior to each move reflect sensitivity to this benefit. Note that we predict that the benefit of computation should influence the time players spend thinking about which move to play. We do not specify how players in practice estimate the benefit of computation, and do not make any hypothesis about whether estimating or computing the benefit of computation itself should be related to move times.

To compute the benefit of computation, we specifically operationalize $m_{-C}$ as the move that Stockfish would select following a depth‐1 search, which involves evaluating moves by applying a neural network static evaluator to the resultant board state (Fig. 1; Low Computation). To approximate the true utility of moves, $U_{C}$, and the optimal move, *m* *c*, we use a depth‐15 search (Fig. 1; High Computation). At this depth, Stockfish plays above grandmaster level (Ferreira, 2013), and we treat its evaluations as ground truth.

Our computation of the benefit of computation involves two choices: using depth‐15 Stockfish as the approximation of true utility, and using depth‐1 Stockfish as an approximation of human play without computation. In the Supplementary Section *Interrogating the validity and necessity of assumptions underlying the benefit of computation*, we evaluate the validity of the assumptions underlying our use of depth‐1 and depth‐15 Stockfish to provide these quantities (Figs. S2 and S3), and also test the robustness of our results against varying these assumptions (Figs. S4–S8). We find that at an aggregate level, our definition of the benefit of computation effectively describes how moves change when players choose to spend more time thinking, either due to greater benefit of computation, or due to having more time available. Additionally, our results remain robust across a range of methods for approximating the effects of computation.

### 5.2 The benefit of computation explains time spent thinking

We used the Stockfish chess engine to estimate the benefit of computation, ∆$U_{C}$, for each board position occurring in 12.5 million games from the Lichess database, spanning the 11 default time‐control settings (see Section 3.2). Using these estimates, we analyzed the relationship between the benefit of computation and the time players spent thinking (indicated by their move times). Across all time‐control settings, we observed a positive relationship between ∆$U_{C}$ and move times, suggesting that the time players’ spend thinking is influenced by the potential benefits of computation (Fig. 3; linear regression predicting move time as a function of ∆$U_{C}$ separately for each time‐condition, with player strength measure Elo included as covariate; estimates between 1.76 and 38.7 s across time‐control settings per increase in 1 $\Delta U_{C}$; all *p*‐values < 1e‐50; see Table S3 for all statistics). The change in move time as a function of the benefit in computation is quite large. For example, in a 60‐s game, the change in mean move time as benefit of computation changes from 0 to .4 is about .67 s. In a 600‐s game, it is about 6.7 s.

Fig. 3 shows that this relationship is concave, with a diminishing slope as ∆$U_{C}$ increases. To test for concavity, we compared linear regression models that predicted move times based on either the benefit of computation, ∆$U_{C}$, or its square root, $\sqrt{\Delta U_{C}}$, and found the latter to better fit the data (ΔAIC=3.6e8; Table S4). Additionally, the relationship between move times and benefit of computation varied with the time‐control setting. In settings with more initial time or a larger increment, move times were longer, and more sensitive to changes in ∆$U_{C}$ (Table S5; linear regression predicting move times from interaction of $\sqrt{\Delta U_{C}}$ and total condition time − Estimate = .02, *p* < 1e‐50 − and interaction of $\sqrt{\Delta U_{C}}$ and increment: Estimate = 1.39, *p* <1e‐50).

### 5.3 Effect of benefit of computation on move times increases with playing strength

If modulation of move time by the benefit of computation is adaptive for efficient planning, we would expect this modulation to increase with greater expertise. To examine this hypothesis, we tested whether the influence of the benefit of computation on move times varies with player Elo, a measure of playing strength. The impact of the benefit of computation on move times increases with player Elo in all time‐control conditions, suggesting that more skilled players use their time more strategically (see example time‐control settings in Fig. S1 and detailed statistics in Table S4; linear regression predicting move time from the interaction of Elo; estimates range from 0.02 to 3.07 across time‐control settings, all *p* < 1e‐50; see Fig. 4 for Spearman correlation between move times and Δ$U_{C}$ as a function of Elo). We acknowledge that the change in the relationship as Elo increases, while significant and robust, is modest.

This analysis relies on using depth‐1 Stockfish as an approximation of human play without computation, which could bias the results if Stockfish more closely mirrors the play style of higher‐rated players. To control for this, we tested an alternative model where $m_{-C}$ is selected by a model specifically trained to mimic the moves of lower‐rated players (McIlroy‐Young et al., 2020). The observed relationships between the intelligent use of time and player Elo were consistent under this alternative model, ruling out these potential biases (Fig. S8).

## 6 Expected benefit of computation explains additional variance in move times

Thus far, we have demonstrated that move times are related to the benefit of computation, ∆$U_{C}$, and that this effect is more pronounced in stronger players. However, a limitation of these findings is that our computation of ∆$U_{C}$ assumes knowledge of the outcome of the computation, $U_{C}$. While this is useful for determining whether computation is beneficial, it does not reflect the evaluation players actually perform: if players knew the outcome in advance, performing the computation would be unnecessary. In the absence of “oracle” knowledge of the results of computation, the principled strategy is to estimate the benefit of computation in *expectation*, by assuming a distribution of outcomes of the computation and computing an estimate that marginalizes over this uncertainty (Keramati et al., 2011; Sezener et al., 2019). In this section, we define a method to estimate the benefit of computation in expectation by assuming a distribution of outcomes of computation. Similar to our previous analysis, we do not claim that players explicitly make this estimation. Instead, we examine whether the situations in which players choose to spend time are consistent with a strategy that acts as if it is based on such intelligent estimation.

### 6.1 The expected benefit of computation

The expected benefit of computation extends the benefit of computation by removing the assumption that players have knowledge of the true (depth‐15) utility of a move $U_{C}\left(m\right)$ in advance. Rather, we assume that at the start of each turn, players have a probabilistic belief over this quantity $P(U_{C}\left(m\right))$ (Fig. 5a). Our specific implementation of this model assumes that this uncertainty is specified by a normal distribution centered on $U_{-C}\left(m\right)$. We make an additional simplifying assumption that players have accurate knowledge of the uncertainty of this distribution and thus set a standard deviation equal to $|U_{C}\left(m\right)-U_{-C}\left(m\right)|$, the absolute difference between $U_{-C}\left(m\right)$ and $U_{C}\left(m\right)$ (note that this is the maximum likelihood estimate for the standard deviation given a mean and a single sample—while we expect this to be true on average, this is not intended as an algorithmic claim about how this uncertainty is estimated). Importantly, this uncertainty is with respect to the result of computation, $U_{C}\left(m\right)$, which is used to compute the benefit of computation, not the benefit of computation itself. While these assumptions about the representation of uncertainty are likely approximations of how people account for uncertainty, these simplifying assumptions allow us to compute the expected benefit of computation directly, and, as we will demonstrate below, allow us to better capture people's behavior.

Given probabilistic beliefs about the true utility of each move, $P(U_{C}\left(m\right)),$ players can then compute the benefit of computation in expectation:

$$E\;\left[\Delta U_{C}\right]=E_{U_{C}}\;\left[U_{C}\left(m_{c}\right)-U_{C}\left(m_{-c}\right)\right]$$

Effectively, computing this quantity involves computing what the benefit of computation, $\Delta \;U_{C}=U_{C}\left(m_{c}\right)-U_{C}\left(m_{-c}\right)$, would be under every possible outcome of computation, $U_{C}$, and then averaging these possible $\Delta U_{C}$, each weighted by $P(U_{C}\left(m\right)).\;$ Given the assumptions made about $P(U_{C}\left(m\right))$, this quantity can be computed efficiently, using Gaussian identities.

As with the benefit of computation, under this setting, computation is still only *actually* valuable when it changes the preferred move (i.e., $m_{c}\neq \;m_{-c}$), and if the new move improves the board position advantage, $U_{C}\left(m_{c}\right)$ > $U_{C}\left(m_{-c}\right)$. However, because $U_{C}$ and $m_{c}$ are not known before performing the computation, the expected value of computation considers whether these conditions are met in expectation. For each possible outome of computation ($U_{C}$ for each move in the consideration set, and resulting $m_{c}$), the resulting benefit is calculated and averaged, weighted by its probability. Intuitively, $E[\Delta U_{C}]$ is higher when there is significant overlap between the distributions $P(U_{C}\left(m\right))$ for different moves, making it valuable to reduce uncertainty before deciding. Conversely, $E[\Delta U_{C}]$ is low when there is little overlap between the distributions $P(U_{C}\left(m\right))$, as reducing uncertainty through computation is unlikely to result in a better move.

To compute the expected benefit of computation $E[\Delta U_{C}],$ we again use a depth‐15 evaluation for $U_{C}$ and define $m_{-C}$ as the move chosen by a depth‐1 search. To make computation of the expectation tractable, we assume that $m_{-C}$ and $m_{C}$ are selected from a consideration set of five moves made up of the top five moves preferred by Stockfish at depth‐1.

### 6.2 Expected benefit of computation explains additional variance in move times

We found that move times increased with greater $E[\Delta U_{C}]$ (estimates between 3.8 and 70.6 s per increase in 1 $E[\Delta U_{C}]$ for different time‐control settings; all *p*‐values < 1e‐50; Table S6). The relationship was again concave (better fit for $\surd E[\Delta U_{C}]$ than $E[\Delta U_{C}]$; ΔAIC=8.9e7; Table S7; Fig. 5b). Moreover, move times were more strongly related to $E[\Delta U_{C}]$ than to $\Delta U_{C}\;$(better fit for $\surd E[\Delta U_{C}]$ than $\surd \Delta U_{C}$; ΔAIC=1.9e8).

Incorporating uncertainty in the benefit of computation thus produces an estimate that aligns more closely with when players spend time thinking. To more transparently assess differences between the ability of $E[\Delta U_{C}]$ and $\Delta U_{C}$ to explain move times, we can look at cases where their predictions diverge. This occurs notably in positions where $\Delta U_{C}=0$ but $E[\Delta U_{C}]$ is positive. For example, when the move with the highest utility, $U_{-C}$, before computation is also the move with the highest utility, $U_{C},$ after computation (i.e., $m_{C}=m_{-C}$), but the player cannot be certain of this equality beforehand (see Fig. 6a for an example). Fig. 6b shows that substantial variation in $E[\Delta U_{C}]$, even when $\Delta U_{C}=0$, accounts for changes in move times (Estimate = 42.5, *p* < 1e‐50). These findings suggest a method by which players might estimate the benefit of computation without knowing the outcomes in advance.

## 7 Move times show signatures of sensitivity to cost of computation

So far, our analysis has focused on the potential benefits of performing computations. However, to decide when a computation is worthwhile, an intelligent strategy must also consider its costs. One significant cost of computations is the time required to execute them, which reduces the time available for future moves. In this section, we examine whether players are sensitive to this time cost by analyzing whether features of when they spend time thinking (Fig. 3) align with the characteristics of a strategy that is sensitive to the actual cost‐structure of spending time in online chess.

### 7.1 Hypothesized effects of cost‐structure of time on move times

A large set of decision‐making tasks, ranging from foraging to evidence accumulation, ask how much time an individual should be willing to wait to obtain some benefit (Agrawal et al., 2022; Garrett & Daw, 2020; Gold & Shadlen, 2002; Keramati et al., 2011; Stephens & Krebs, 1986). In the prey selection task, for example, an individual must decide whether to accept or reject prey based on varying benefits (calories) and the time required to obtain them. The optimal strategy is to accept prey if the time required, ΔT, is less than a threshold $\Delta T_{\max}$, where $\Delta T_{\max}$ is the value at which the benefit equals the cost of time, c(ΔT). An insight from this and related tasks is that the shape these decision policies—how $\Delta T_{\max}$ varies with the magnitude of the offered benefit— depends on the cost‐structure of time (Drugowitsch et al., 2012; Steverson, Chung, Zimmermann, Louie, & Glimcher, 2019).

To explore how move times would indicate sensitivity to the cost of time, we make an analogy to such tasks and consider a simplified setting. In this scenario, the player has a current board position advantage, U, and an amount of time remaining, T. The player is offered a computation, $C=(\Delta T_{C},\Delta U_{C})$, where $\Delta T_{C}$ is the time cost which decrements their clock, $T\;\leftarrow \;T\;-\;\Delta T_{C}$, and $\Delta U_{C}$ is the benefit to board position, $U\;\leftarrow \;U+\Delta U_{C}$. For simplicity, we use the true benefit rather than the expected benefit. We consider the maximum amount of time a player should be willing to spend to achieve a certain benefit based on different cost‐structures. While this problem is a simplification compared to the full problem of deciding how long to think, it can still be used to assess whether the observed relationship between move times, computation benefits, and time‐control aligns with an intelligent consideration of the time cost in online chess.

**Fig. 7:**
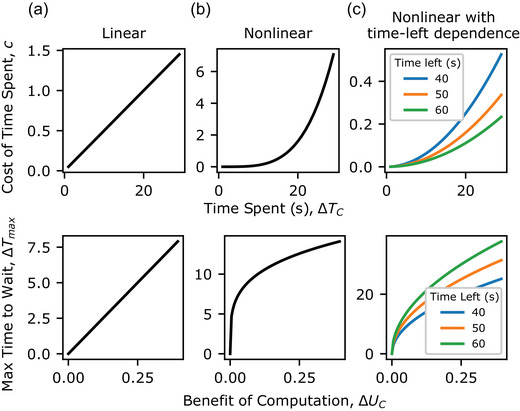
Hypothesized cost‐structures for spending time in online chess and corresponding optimal policies. (a) Linear increasing cost‐structure. Linear increasing cost over time spent supports optimal policies where time spent increases linearly with benefit. (b) Nonlinear increasing cost‐structure. Nonlinear increasing cost over time spent supports optimal policies where time spent increases linearly with benefit, where time spent increases in a concave manner with benefit. (c) Nonlinear increasing with time‐left dependence cost‐structure. Nonlinear increasing cost over time spent with time‐left dependence cost‐structure supports optimal policies where time spent increases linearly with benefit where time spent increases in a concave manner with benefit, and also depends on time left.

We consider several classes of hypothetical cost‐structures, and their corresponding optimal policies (Fig. 7). In the most typical framing of such tasks, the objective is to maximize average reward per unit time, leading to a linear increase in the cost of time with the duration spent, $c\;\left(\Delta T_{C}\right)=\rho \Delta T_{C,}$ where ρ is the opportunity cost per unit time‐step (Bertsekas, 2012; Stephens & Krebs, 1986). In this case, the maximum time, $\Delta T_{max}$, increases linearly with the benefit $\Delta U_{C}$ $(\Delta T_{max}=\frac{1}{\rho }\;\Delta U_{C}$ Fig. 7a).

However, different objectives specify different cost‐structures and optimal policies. For example, if the cost of time increases nonlinearly with time spent (e.g., $c\;\left(\Delta T_{C}\right)=a\Delta T_{C}^{b}$, where a>0 and b>1), then the time the optimal policy would be willing to spend increases in a concave manner with increasing benefit ($\Delta \;T_{max}={\left(\frac{1}{a}\Delta U_{C}\right)}^{\frac{1}{b}}\;$; Fig. 7b). In chess, where players lose once their clock runs out, the cost function may also depend on time remaining at the start of a decision, *T*. Fig. 7c shows one such example, $c\left(\Delta T_{C}\right)=a{\left(\frac{\Delta T_{C}}{T}\right)}^{b}$. In such a case, the optimal policy is both concave and depends on the time left at the start, $\Delta T_{max}=T{\left(\frac{\Delta U_{C}}{a}\right)}^{\frac{1}{b}}$.

Our measurement of the relationship between move times and the benefit of computation (Fig. 3) revealed that move times increase concavely with increasing benefit of computation and vary with time‐control setting. Because time‐control setting dependencies could reflect time‐left dependencies (because in games with greater time, more moves occur with more time left), these results might suggest that players’ policies over move times intelligently reflect a cost‐structure that increases nonlinearly with time spent and depends on the time remaining. We further investigated this by measuring the actual cost‐structure of spending time in online games.

### 7.2 Measuring the cost‐structure of spending time

**Fig. 8:**
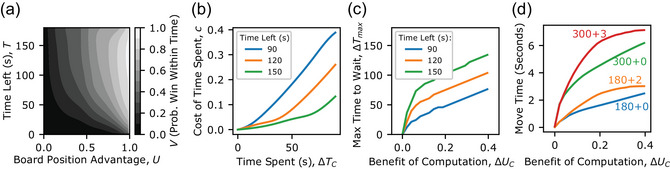
Empirically measured cost‐structure and trade‐off with benefit of computation can explain qualitative features of player's observed move times. (a) Empirical value function over time‐left and board position advantage. We computed $V_{tc}\left(T,U\right)$ as the empirical probability of winning a match following for time‐condition *tc*, following a game state with time‐left T and board position advantage U. Image shows $V_{tc}$ from 180+0 time‐control setting. (b) Empirically measured cost‐structure. The approximate cost of spending time, *c*(*ΔT*), can be measured as the decrease in $V_{tc}\left(T,U\right)$ as T decreases by ΔT. The measured cost function is similar to the hypothesized nonlinear with time‐left dependence cost‐structure. Here, U is set to .5. (c) Optimal policy implied by empirically measured cost‐structure. For a given benefit, $\Delta U_{C}$, $\Delta T_{max}$ is computed as the maximum $\Delta T_{C}$ such that performing computation is still worthwhile, $V_{tc}\left(T-\Delta T_{C},U+\Delta U\right)\geq V\left(T,U\right)$. (d) Mean move times implied by empirically measured cost‐structure. Mean move times for optimal policy under the empirically measured cost‐structure were computed over the empirically estimated distribution of computational offers. Features of computed mean move times demonstrate that the empirically measured cost‐structure can underlie the concave increase in move times with the benefit of computation, and dependence of move times on both total clock time and increment. A subset of time‐control settings are displayed to enable visualization of key qualitative effects.

In chess, the cost of spending time can be measured as the reduction in a player's chances of winning as they use up their clock time. To measure this cost‐structure, we calculated for each time‐control setting, tc, an empirical value function, $V_{tc}\left(T,U\right)$ (Fig. 8a; see Fig. S10 for all time‐control settings). This function estimates the probability of winning the game before running out of time, given a game state with T s remaining and board position advantage U.

To compute $V_{tc}\left(T,U\right),$ we split moves into bins specified by the time left (3 s intervals), and board position advantage (.03 Utility intervals). Note that in defining bins, we defined Utility it to be the value of the resultant board position, if the agent were to not execute the computation, $\Delta U_{-C}$. Thus, offered changes along this dimension, $\Delta U_{C}$ reflect the increase to board position advantage from executing a computation. $V_{tc}\left(T,U\right)$ was then computed as the proportion of moves in each bin where the active player won. Note that we use the phrase empirical to refer to the fact that each of these proportions is measured empirically (as the fraction of moves in the dataset where the active player won), as opposed to being estimated from a prespecified functional form or assumed relationship between states.

A challenge in examining changes in T is that it is often correlated with the opponent's time left. To isolate the impact of time left independently of the opponent's time, we limited our analysis to moves where the opponent had at least 60 s remaining (or 30 s for the 60+0 time‐control). Note that $V_{tc}\left(T,U\right)$ is independent of the actual time that a player spent thinking in a position. Additionally, while we use Stockfish evaluation to place moves into bins for different U (characterizing which player is winning and by how much), the function $V_{tc}\left(T,U\right)$ reflects probabilities computed empirically from human games and thus is not dependent on engine evaluation. Changes in $V_{tc}\left(T,U\right)$ with varying T and U reflect estimates as to how human Lichess players’ winning chances are expected to shift as clock time and board position advantage change.

Given $V_{tc}$, the cost of spending time, $c(\Delta T_{C\;}),$ can be approximated as the extent to which $V_{tc}\left(T,U\right)$ decreases as T is decremented by $\Delta T_{C}$, $c(\Delta T_{C})\;\approx \;V_{\mathrm{tc}}(T,U)\;-\;V_{\mathrm{tc}}(T\;-\;\Delta T_{C},U).$ Fig. 8b shows that $c(\Delta T_{C})$ increases nonlinearly with time spent and depends on time remaining, supporting the proposal that players’ policies may reflect sensitivity to the true cost‐structure of spending time. We again note that this measure of the cost of time is completely independent of any measurement of move times, and does not reflect the fit of any particular model. Rather, this simply reflects empirically how win‐rates in online chess games decrease as time is used up.

To validate that this empirically measured cost‐structure implies optimal move times that are concave and dependent on time left, we used the empirically measured value function to compute, for each potential benefit of computation, $\Delta U_{C}$, the maximal amount of time, $\Delta T_{C}$, one could spend, such that performing computation would still be worthwhile, $\Delta T_{\max}=\Delta T_{C}\;s.t.\;V_{\mathrm{tc}}(T\;-\;\Delta T_{C},U+\Delta U_{C})\;\geq \;V(T,U)$. As predicted based on the cost‐structure's features, these computed $\Delta T_{max}$ values increas concavely with $\Delta U_{C}$, and depend on time left (Fig. 8c). We again note that these curves are in no sense “fit” to move‐time data, but rather are generated purely from measurement of how win‐rates change with respect to changes in time and board position advantage.

Finally, we confirmed that a policy based on $\Delta T_{\max}$ would produce mean move times, for each benefit of computation and time‐control, resembling the relationship observed in Fig. 3. We note that this exercise is not intended to provide a model of the generation of move times, but rather is meant to explain the qualitative pattern with which move times vary with the benefit of computation. For each time‐control and ∆$U_{C}$, we computed the mean time this policy would spend by defining a distribution over starting states (T,U) and offered computation times $\Delta T_{C}$. Whereas the distribution for (T,U) could be derived from Lichess data, a distribution over $\Delta T_{C}$ was approximated using the time Stockfish took to reach the move $m_{c}$, from sampled positions, and multiplying this by a free scaling parameter, s, set to 20,000 for the results presented here. Based on this distribution, we calculated the expected time the optimal policy (Fig. 8c) would spend by determining the time allocation for each combination of computation offer $(\Delta T_{C},\Delta U_{C}),$ current board position advantage U, and remaining clock time T, and then marginalizing over these distributions. The mean move times of the implied policy capture key characteristics of the observed relationship between move times and the benefit of computation (Fig. 8d; see Fig. S11 for all time‐control settings). Like the observed move times, the implied move times increase concavely with the benefit of computation. Moreover, they reflect a dependency not only on total clock time but also on time increments, indicating that these patterns align with sensitivity to the cost of time. This suggests that the measured relationship between move times and the benefit of computation indeed reflects sensitivity to the cost‐structure of spending time in online chess.

Notably, the relationship between move times and time‐control settings for the implied policy is driven by a dependence on time remaining, T. The implied policy for the measured cost‐structure suggests that faster moves should be made as T decreases (Fig. S12). Conversely, when conditioned on T, U, and $\Delta U_{C}$, the implied policy's move times remain unchanged across different time‐control settings (Fig. S13). In contrast, human move times were not as strongly modulated by T. Furthermore, the change observed was nonmonotonic: the slowest moves occurred at intermediate values of T, with faster moves occurring both at the start and the end of the game. This discrepancy suggests that the estimated benefit of computation early in a game may be lower than our model suggests, possibly due to differences in how humans and Stockfish decide on moves at the start of games. Humans likely predecide on openings, unlike Stockfish, leading to discrepancies in the estimated benefit of computation. Additionally, human move times were influenced by the time‐control setting, even controlling for T, U, and $\Delta U_{C}$. This suggests that human move times align with the optimal policy's predictions for time left, but only in aggregate across time‐control settings, and may deviate within each setting. This discrepancy could result from specific heuristic strategies, where players store and reuse the average time spent by the optimal policy for each ∆$U_{C}$ separately for each time‐control setting. However, the precise investigation of specific heuristics by which players approximate an optimal trade‐off is left for future work.

## 8 Discussion

Recent theoretical frameworks have suggested that a core component of human cognition is the ability to intelligently use limited resources to decide (Boureau et al., 2015; Gershman et al., 2015; Lewis et al., 2014; Lieder & Griffiths, 2020; Shenhav et al., 2013). Here, we investigated this proposal in the domain of complex planning by examining how players spend time in online chess games in a massive dataset. Specifically, we identified a relationship between the benefit of computation and time spent thinking across different board positions. This relationship was stronger in higher‐skilled players and was improved by incorporating uncertainty—reflecting knowledge individuals would have at the time of computation—into estimates of the benefits of computation. Finally, characteristics of players’ move times reflected the incorporation of the cost‐structure of spending time in online chess. Together, our findings demonstrate that the time that players spend thinking shows signatures of sensitivity to the benefits and costs of computation, and that this sensitivity increases with expertise.

Measuring the benefit of computation requires a model of how computation alters choices. In our primary approach, we considered that computation can provide the optimal move and measured the difference in utility between this and a move selected without computation, approximated by depth‐1 Stockfish. Although this model is an approximation of human computation in chess, we believe this approximation is sufficient to support the analysis of this study. In addition to validating that this approximation captures, in aggregate, how human moves change with move time increases induced by benefit of computation and time‐control, we also found that our key results were robust to changing this approximate model of computation to include a wider variety of approaches to move selection, evaluation, as well as different engines (see Supplementary Section *Interrogating the validity and necessity of assumptions underlying the benefit of computation*). This robustness to a variety of approaches suggests that what our measured benefit of computation captures is not specific to computation by Stockfish, but rather extends toward more general decision‐tree search. A range of work modeling human decisions in multistep planning tasks (Huys et al., 2012, 2015), multiplayer board games (van Opheusden et al., 2021), and also using think out‐loud paradigms in chess (Campitelli & Gobet, 2004; De Groot, 1946; Saariluoma, 1992) suggests that human computation utilizes similar search processes, thus supporting the validity of our approximation.

Our approach treats computation as an all‐or‐none discrete computational action that provides the true utility of all moves under consideration. This framing is similar to prior modeling of the model‐based and model‐free trade‐off in decision‐making, where an agent decides whether to use cached model‐free action valuations or model‐based reasoning (Daw, Niv, & Dayan, 2005; Keramati et al., 2011; Kool et al., 2017). Although this all‐or‐none model of computation makes our analysis tractable, this is not intended to be a substantive claim about how human computation works or is evaluated. Rather, it is likely that planning in chess is an incremental process, and that the decision about how long to plan makes use of partial computations made thus far into deliberation (Agrawal et al., 2022; Mattar & Daw, 2018; Sezener et al., 2019). Modeling the decision of how long to continue planning at this level will likely require a more fine‐grained model of how humans plan in chess, which we view as an important challenge for future work.

Our initial approach to computing the value of computation used a form of oracle knowledge about the results of computation—the post‐computation utility of moves. Our methods for computing the expected value of computation, which takes a step toward removing this oracle knowledge, still used a depth‐15 evaluation to compute the uncertainty around the estimate of the resultant post‐computation utility function. This was not intended as an algorithmic claim for how individuals estimate the error of their precomputation estimates of move utility. We anticipate that future work can explore how feature‐based approximate predictions of estimate error could be learned, in a manner similar to how neural networks are currently used to predict position utility. A similar approach, proposed in recent work, could potentially also support learning predictions of the benefit of computation itself, in a model‐free manner (Lieder & Griffiths, 2017). Supporting this idea, work in cognitive control has demonstrated that model‐free learning may be used to learn appropriate mental effort (Grahek et al., 2023).

While our measurement of the true cost‐structure of spending time in chess revealed that sensitivity to this cost would produce move times that increase in a concave manner with the benefit of computation, we note that other possible explanations for this qualitative effect exist. In many tasks, response times increase logarithmically with either the number of choice options or choice difficulty, a phenomenon that was formalized with Hick's law (Hick, 1952). To the extent these measures increase with the benefit of computation, they may induce a concave increase. We note, however, an advantage of the cost of time explanation is its additional ability to explain effects of both total time and increment features of time‐control setting on move times.

Finally, although our findings provide evidence that human move times show signatures of sensitivity to the benefits and costs of computation, we have not provided evidence of the specific process that players follow to achieve such sensitivity. While we have tested a computational‐level hypothesis based on the rational metareasoning framework, we have not specified what algorithms are responsible for achieving this computational objective. Future work should test specific process‐level hypothesis for how sensitivity to the value of computation is achieved. This work can seek to disentangle whether processes that players engage in either explicitly estimate the expected benefits and costs of computation, for example, by feature‐based estimation (Lieder & Griffiths, 2017), or alternatively utilize heuristic strategies, possibly related to those suggested in the chess literature (Baier & Winands, 2016; Beal, 1990; Burduli & Wu, 2023; Donninger, 1994; Hay et al., 2012; Huang et al., 2010; Hyatt, 1984; Kocsis et al., 2001; Markovitch & Sella, 1996; Newell & Simon, 1972; Turing, 1953; Vuckovic & Solak, 2009) that might behave as though they perform such an estimation.

Our results dovetail with other studies that have recently investigated how much time humans spend when planning chess moves. A recent study of move times in a dataset of professional chess games, smaller than that we present here, found relationships with time remaining on a player's clock, the difference in value between the best and second‐best move, and position complexity (Sunde et al., 2022). Recent work has also trained feature‐based estimators to predict move times on chess.com (Burduli & Wu, 2023). While the attributes used to predict response times in both these studies may relate to the benefits and costs of computation, they differ from the approach that we take here, which involves an actual quantification of those costs and benefits with the aim of determining how they relate to players’ behavior and how this relationship changes with expertise.

In addition to demonstrating that the sensitivity of thinking to benefit of cognition extends to a large‐scale naturalistic domain, our findings bear on two additional threads in cognitive science. First, while the study of expertise in cognitive domains has so far largely focused on either improvement to search or pattern recognition (Bilalić, McLeod, & Gobet, 2008; Campitelli & Gobet, 2004; Charness, 1981; Chase & Simon, 1973; De Groot, 1946; Van Harreveld, Wagenmakers, & Van Der Maas, 2007; van Opheusden et al., 2021), here we demonstrate that improvements to policies about when to spend limited cognitive resources also form a key component. Additionally, whereas much of the research on opportunity cost of time has used the average reward rate as a measure of this (Agrawal et al., 2022; Gold & Shadlen, 2002; Niv et al., 2007; Otto & Daw, 2019), here we demonstrate a domain where opportunity cost of time has a different functional form, and human move times adapt accordingly. This finding thus shows additional flexibility in how humans can represent the time‐based costs of deciding.

By demonstrating that human thinking times balance a trade‐off between the benefits of computation and the cost of time spent, we have contributed part of an explanation for the efficiency of human cognition in large problems like chess. Expanding this framework from *when* to compute to *what* areas to focus computation on, and also addressing the types of representations used during planning, is likely to yield additional insights into understanding how humans plan so efficiently. Large datasets that can allow precise testing of computational hypotheses are likely to play an important role in the further development of this work.

## Code availability statement

Analysis code is publicly available at https://github.com/evanrussek/Thinking_Time_VOC_Chess.

## Supporting information



Supporting Information

## Data Availability

All data analyzed are publicly available on the lichess.org, database, https://database.lichess.org/. We analyzed a subset of this data, utilized for the Maia Chess Project, which is formatted into CSVs and available here: http://csslab.cs.toronto.edu/datasets/#monthly_chess_csv.
