# Supplementary material for: Time Spent Thinking in Online Chess Reflects the Value of Computation
Source: Cogn Sci. 2025 Oct 25;49(10):e70119. doi: 10.1111/cogs.70119 (PMC12553403; doi:10.1111/cogs.70119)
Supplement: Supplementary file 1 — Supporting Information [file COGS-49-e70119-s001.docx]

Supplementary Tables and Figures

**Additional statistics for all analysis**

**Supplementary Table 1.** Number of moves and games from each time-control setting used for analysis presented in Figs. 2, 3 and 6. Note that due to low number of games, 1800+20 was not included in Fig. 6.

|  | **Number of Moves** | **Number of Games** |
| --- | --- | --- |
| **60+0** | **95735227** | **2308512** |
| **120+1** | **27051987** | **663731** |
| **180+0** | **75861368** | **1782268** |
| **180+2** | **43048859** | **1032088** |
| **300+0** | **96356402** | **2318583** |
| **300+3** | **52568904** | **1283127** |
| **600+0** | **123569358** | **2980966** |
| **600+5** | **2138919** | **49478** |
| **900+10** | **1403375** | **32488** |
| **1800+0** | **1601731** | **37774** |
| **1800+20** | **42706** | **1031** |

**Supplementary Table 2. Number of moves and games from each time-control setting used** for analysis presented in Fig. 4

|  | **Number of Moves** | **Number of Games** |
| --- | --- | --- |
| **60+0** | **33981700** | **800012** |
| **120+1** | **10823685** | **256853** |
| **180+0** | **34443968** | **779972** |
| **180+2** | **17158527** | **399728** |
| **300+0** | **44362458** | **1040103** |
| **300+3** | **21902577** | **522953** |
| **600+0** | **56436648** | **1323328** |
| **600+5** | **845402** | **19518** |
| **900+10** | **553048** | **12432** |
| **1800+0** | **557543** | **13192** |
| **1800+20** | **14548** | **453** |

**Supplementary Table 3.** Results of linear regression model predicting move time as function of benefit of computation, $\Delta U_{C}$ (listed above as **ΔUC),** player ELO and their interaction, with a separate regressor for each time-control setting. Note that player ELO was centred at 1500.

|  | **Estimate** | **Std. Error** | **z** | **P** |
| --- | --- | --- | --- | --- |
| **60+0** | 1.762207 | 0.001102 | 1598.983193 | 0.000000 |
| **120+1** | 4.270410 | 0.002096 | 2036.998439 | 0.000000 |
| **180+0** | 4.319600 | 0.001371 | 3151.011916 | 0.000000 |
| **180+2** | 6.525063 | 0.001687 | 3868.725559 | 0.000000 |
| **300+0** | 6.745019 | 0.001105 | 6101.471818 | 0.000000 |
| **300+3** | 10.163488 | 0.001570 | 6475.331398 | 0.000000 |
| **600+0** | 11.429693 | 0.000976 | 11715.714806 | 0.000000 |
| **600+5** | 15.603269 | 0.008557 | 1823.352770 | 0.000000 |
| **900+10** | 23.614238 | 0.010499 | 2249.119104 | 0.000000 |
| **1800+0** | 23.773335 | 0.009607 | 2474.551350 | 0.000000 |
| **1800+20** | 38.739424 | 0.068029 | 569.453621 | 0.000000 |
| **60+0 ELO** | -0.056641 | 0.000312 | -181.672309 | 0.000000 |
| **120+1 ELO** | -0.042023 | 0.000646 | -65.067024 | 0.000000 |
| **180+0 ELO** | -0.070395 | 0.000337 | -208.967900 | 0.000000 |
| **180+2 ELO** | -0.008220 | 0.000485 | -16.935998 | 0.000000 |
| **300+0 ELO** | -0.102383 | 0.000331 | -309.683137 | 0.000000 |
| **300+3 ELO** | 0.028162 | 0.000522 | 53.972216 | 0.000000 |
| **600+0 ELO** | -0.086068 | 0.000327 | -262.810426 | 0.000000 |
| **600+5 ELO** | 0.411091 | 0.002678 | 153.498333 | 0.000000 |
| **900+10 ELO** | 0.766817 | 0.003684 | 208.122912 | 0.000000 |
| **1800+0 ELO** | 0.936312 | 0.003870 | 241.910499 | 0.000000 |
| **1800+20 ELO** | 2.474405 | 0.023712 | 104.353486 | 0.000000 |
| **60+0 ΔUC** | 2.647601 | 0.021075 | 125.628599 | 0.000000 |
| **120+1 ΔUC** | 8.647010 | 0.033093 | 261.293472 | 0.000000 |
| **180+0 ΔUC** | 8.892451 | 0.022830 | 389.504200 | 0.000000 |
| **180+2 ΔUC** | 13.206403 | 0.026767 | 493.382435 | 0.000000 |
| **300+0 ΔUC** | 14.188941 | 0.017369 | 816.919193 | 0.000000 |
| **300+3 ΔUC** | 21.011259 | 0.023899 | 879.152021 | 0.000000 |
| **600+0 ΔUC** | 24.387395 | 0.015426 | 1580.964494 | 0.000000 |
| **600+5 ΔUC** | 35.225212 | 0.139550 | 252.419552 | 0.000000 |
| **900+10 ΔUC** | 49.640609 | 0.168836 | 294.016666 | 0.000000 |
| **1800+0 ΔUC** | 52.458719 | 0.157341 | 333.408630 | 0.000000 |
| **1800+20 ΔUC** | 97.634283 | 1.186856 | 82.262971 | 0.000000 |
| **60+0 ELO & ΔUC** | 0.023219 | 0.005586 | 4.156686 | 0.000032 |
| **120+1 ELO & ΔUC** | 0.186577 | 0.010216 | 18.262582 | 0.000000 |
| **180+0 ELO & ΔUC** | 0.278008 | 0.005304 | 52.412550 | 0.000000 |
| **180+2 ELO & ΔUC** | 0.397620 | 0.007486 | 53.113102 | 0.000000 |
| **300+0 ELO & ΔUC** | 0.382024 | 0.005179 | 73.761818 | 0.000000 |
| **300+3 ELO & ΔUC** | 0.631274 | 0.008116 | 77.784844 | 0.000000 |
| **600+0 ELO & ΔUC** | 0.841599 | 0.005111 | 164.674377 | 0.000000 |
| **600+5 ELO & ΔUC** | 1.197148 | 0.041644 | 28.746955 | 0.000000 |
| **900+10 ELO & ΔUC** | 2.265766 | 0.056632 | 40.008439 | 0.000000 |
| **1800+0 ELO & ΔUC** | 4.528462 | 0.061115 | 74.097302 | 0.000000 |
| **1800+20 ELO & ΔUC** | 1.533860 | 0.391344 | 3.919469 | 0.000089 |

**Supplementary Table 4.** Results of linear regression model predicting move time as function of square root of benefit of computation, $\surd\Delta U_{C}$ (listed above as ΔUC), player ELO and their interaction, with a separate regressor for each time-control setting. Note that player ELO was centered at 1500.

|  | **Estimate** | **Std. Error** | **z** | **P** |
| --- | --- | --- | --- | --- |
| **60+0** | 1.713425 | 0.001250 | 1370.553540 | 0.000000 |
| **120+1** | 4.060704 | 0.002377 | 1707.989231 | 0.000000 |
| **180+0** | 4.117303 | 0.001551 | 2654.235767 | 0.000000 |
| **180+2** | 6.184004 | 0.001909 | 3240.046264 | 0.000000 |
| **300+0** | 6.390903 | 0.001254 | 5098.399678 | 0.000000 |
| **300+3** | 9.592819 | 0.001781 | 5386.779565 | 0.000000 |
| **600+0** | 10.799439 | 0.001105 | 9770.034955 | 0.000000 |
| **600+5** | 14.700288 | 0.009709 | 1514.139994 | 0.000000 |
| **900+10** | 22.268163 | 0.011923 | 1867.656258 | 0.000000 |
| **1800+0** | 22.392348 | 0.010917 | 2051.129515 | 0.000000 |
| **1800+20** | 36.654352 | 0.077076 | 475.561906 | 0.000000 |
| **60+0 ELO** | -0.057768 | 0.000354 | -163.323250 | 0.000000 |
| **120+1 ELO** | -0.051098 | 0.000732 | -69.798827 | 0.000000 |
| **180+0 ELO** | -0.080221 | 0.000382 | -210.085732 | 0.000000 |
| **180+2 ELO** | -0.026911 | 0.000550 | -48.952771 | 0.000000 |
| **300+0 ELO** | -0.115364 | 0.000375 | -307.981158 | 0.000000 |
| **300+3 ELO** | 0.000436 | 0.000591 | 0.737122 | 0.461048 |
| **600+0 ELO** | -0.114329 | 0.000371 | -308.166474 | 0.000000 |
| **600+5 ELO** | 0.334691 | 0.003042 | 110.007949 | 0.000000 |
| **900+10 ELO** | 0.638272 | 0.004189 | 152.360907 | 0.000000 |
| **1800+0 ELO** | 0.779132 | 0.004400 | 177.057640 | 0.000000 |
| **1800+20 ELO** | 2.178966 | 0.026864 | 81.110892 | 0.000000 |
| **60+0 ΔUC** | 1.205023 | 0.008505 | 141.677682 | 0.000000 |
| **120+1 ΔUC** | 4.447850 | 0.014439 | 308.036419 | 0.000000 |
| **180+0 ΔUC** | 4.465719 | 0.009834 | 454.097978 | 0.000000 |
| **180+2 ΔUC** | 7.015774 | 0.011692 | 600.071904 | 0.000000 |
| **300+0 ΔUC** | 7.423948 | 0.007629 | 973.103193 | 0.000000 |
| **300+3 ΔUC** | 11.391193 | 0.010560 | 1078.727793 | 0.000000 |
| **600+0 ΔUC** | 12.996260 | 0.006772 | 1919.004038 | 0.000000 |
| **600+5 ΔUC** | 18.514061 | 0.060238 | 307.349033 | 0.000000 |
| **900+10 ΔUC** | 26.707139 | 0.073121 | 365.243621 | 0.000000 |
| **1800+0 ΔUC** | 27.860244 | 0.067782 | 411.028304 | 0.000000 |
| **1800+20 ΔUC** | 46.772182 | 0.486147 | 96.209926 | 0.000000 |
| **60+0 ELO & ΔUC** | 0.014365 | 0.002316 | 6.201824 | 0.000000 |
| **120+1 ELO & ΔUC** | 0.122142 | 0.004455 | 27.419889 | 0.000000 |
| **180+0 ELO & ΔUC** | 0.155010 | 0.002326 | 66.630784 | 0.000000 |
| **180+2 ELO & ΔUC** | 0.258964 | 0.003299 | 78.504558 | 0.000000 |
| **300+0 ELO & ΔUC** | 0.194055 | 0.002282 | 85.049871 | 0.000000 |
| **300+3 ELO & ΔUC** | 0.384997 | 0.003572 | 107.775183 | 0.000000 |
| **600+0 ELO & ΔUC** | 0.438914 | 0.002257 | 194.479855 | 0.000000 |
| **600+5 ELO & ΔUC** | 0.993115 | 0.018314 | 54.228315 | 0.000000 |
| **900+10 ELO & ΔUC** | 1.747573 | 0.025013 | 69.866358 | 0.000000 |
| **1800+0 ELO & ΔUC** | 2.658690 | 0.026745 | 99.410320 | 0.000000 |
| **1800+20 ELO & ΔUC** | 3.078191 | 0.162387 | 18.955852 | 0.000000 |

**Supplementary Table 5**. Results of linear regression model predicting move time as function of square root of benefit of computation, $\surd\Delta U_{C}$ (listed above as ΔUC), player ELO, clock start time, interacted with clock start time (Total Time), the amount of time back following each move (Time Back).

|  | **Estimate** | **Std. Error** | **z** | **P** |
| --- | --- | --- | --- | --- |
| **Intercept** | 1.299205 | 0.000976 | 1330.818801 | 0.000000 |
| **ELO** | -0.058236 | 0.000282 | -206.175118 | 0.000000 |
| **ΔUC** | 1.185798 | 0.006331 | 187.294968 | 0.000000 |
| **Total Time** | 0.015650 | 0.000003 | 6131.787881 | 0.000000 |
| **Time Back** | 1.054729 | 0.000463 | 2278.239151 | 0.000000 |
| **ELO & ΔUC** | -0.058764 | 0.001779 | -33.023981 | 0.000000 |
| **ELO & Total Time** | -0.000123 | 0.000001 | -148.445195 | 0.000000 |
| **ELO & Time Back** | 0.021290 | 0.000148 | 143.757569 | 0.000000 |
| **ΔUC & Total Time** | 0.019406 | 0.000016 | 1203.653124 | 0.000000 |
| **ΔUC & Time Back** | 1.272612 | 0.002825 | 450.510544 | 0.000000 |
| **ELO & ΔUC & Total Time** | 0.000866 | 0.000005 | 169.099248 | 0.000000 |
| **ELO & ΔUC & Time Back** | 0.053149 | 0.000897 | 59.279523 | 0.000000 |

**Supplementary Table 6.** Results of linear regression model predicting move time as function of expected benefit of computation, $E[\Delta U_{C}]$ (listed above as EΔUC), player ELO and their interaction, with a separate regressor for each time-control setting. Note that player ELO was centered at 1500.

|  | **Estimate** | **Std. Error** | **z** | **P** |
| --- | --- | --- | --- | --- |
| **60+0** | 1.670773 | 0.002225 | 750.887344 | 0.000000 |
| **120+1** | 3.837672 | 0.004048 | 948.017941 | 0.000000 |
| **180+0** | 3.880410 | 0.002486 | 1560.616433 | 0.000000 |
| **180+2** | 5.835061 | 0.003186 | 1831.703732 | 0.000000 |
| **300+0** | 6.042823 | 0.001980 | 3052.635692 | 0.000000 |
| **300+3** | 9.072677 | 0.003022 | 3002.648814 | 0.000000 |
| **600+0** | 10.270236 | 0.001748 | 5875.703270 | 0.000000 |
| **600+5** | 13.685840 | 0.015394 | 889.016453 | 0.000000 |
| **900+10** | 20.638053 | 0.019547 | 1055.814455 | 0.000000 |
| **1800+0** | 21.818958 | 0.018693 | 1167.245959 | 0.000000 |
| **1800+20** | 27.902058 | 0.114897 | 242.843686 | 0.000000 |
| **60+0 ELO** | -0.066667 | 0.000613 | -108.731296 | 0.000000 |
| **120+1 ELO** | -0.061900 | 0.001252 | -49.429624 | 0.000000 |
| **180+0 ELO** | -0.104398 | 0.000608 | -171.760606 | 0.000000 |
| **180+2 ELO** | -0.046837 | 0.000954 | -49.108976 | 0.000000 |
| **300+0 ELO** | -0.166643 | 0.000590 | -282.553139 | 0.000000 |
| **300+3 ELO** | -0.046463 | 0.000985 | -47.183867 | 0.000000 |
| **600+0 ELO** | -0.181446 | 0.000589 | -308.095412 | 0.000000 |
| **600+5 ELO** | 0.014888 | 0.005316 | 2.800924 | 0.005096 |
| **900+10 ELO** | 0.312990 | 0.007369 | 42.471156 | 0.000000 |
| **1800+0 ELO** | 0.652015 | 0.008481 | 76.880973 | 0.000000 |
| **1800+20 ELO** | -4.838885 | 0.104556 | -46.280169 | 0.000000 |
| **60+0 EΔUC** | 3.968375 | 0.040907 | 97.010274 | 0.000000 |
| **120+1 EΔUC** | 14.490207 | 0.065533 | 221.113976 | 0.000000 |
| **180+0 EΔUC** | 15.060851 | 0.041682 | 361.323168 | 0.000000 |
| **180+2 EΔUC** | 21.820227 | 0.051339 | 425.025105 | 0.000000 |
| **300+0 EΔUC** | 24.687701 | 0.031809 | 776.126455 | 0.000000 |
| **300+3 EΔUC** | 34.195978 | 0.047589 | 718.565704 | 0.000000 |
| **600+0 EΔUC** | 40.654561 | 0.028199 | 1441.690186 | 0.000000 |
| **600+5 EΔUC** | 54.867687 | 0.248413 | 220.872645 | 0.000000 |
| **900+10 EΔUC** | 80.279053 | 0.311912 | 257.376868 | 0.000000 |
| **1800+0 EΔUC** | 73.999330 | 0.313967 | 235.691129 | 0.000000 |
| **1800+20 EΔUC** | 90.065006 | 2.287912 | 39.365595 | 0.000000 |
| **60+0 ELO & EΔUC** | 0.167016 | 0.010932 | 15.278338 | 0.000000 |
| **120+1 ELO & EΔUC** | 0.471007 | 0.020399 | 23.089767 | 0.000000 |
| **180+0 ELO & EΔUC** | 0.667618 | 0.009866 | 67.669579 | 0.000000 |
| **180+2 ELO & EΔUC** | 0.938333 | 0.015183 | 61.799566 | 0.000000 |
| **300+0 ELO & EΔUC** | 1.131461 | 0.009501 | 119.091419 | 0.000000 |
| **300+3 ELO & EΔUC** | 1.044142 | 0.015859 | 65.837289 | 0.000000 |
| **600+0 ELO & EΔUC** | 2.018244 | 0.009444 | 213.697372 | 0.000000 |
| **600+5 ELO & EΔUC** | 6.967864 | 0.085024 | 81.951469 | 0.000000 |
| **900+10 ELO & EΔUC** | 6.315419 | 0.110854 | 56.970529 | 0.000000 |
| **1800+0 ELO & EΔUC** | 0.679838 | 0.140219 | 4.848394 | 0.000001 |
| **1800+20 ELO & EΔUC** | -32.845786 | 2.133798 | -15.393113 | 0.000000 |

**Supplementary Table 7.** Results of linear regression model predicting move time as function of square root of benefit of computation, $\surd E[\Delta U_{C}]$ (listed above as EΔUC), player ELO and their interaction, with a separate regressor for each time-control setting. Note that player ELO was centered at 1500.

|  | **Estimate** | **Std. Error** | **z** | **P** |
| --- | --- | --- | --- | --- |
| **60+0** | 1.713404 | 0.002084 | 822.315712 | 0.000000 |
| **120+1** | 4.010690 | 0.003755 | 1068.108636 | 0.000000 |
| **180+0** | 4.092126 | 0.002307 | 1773.637793 | 0.000000 |
| **180+2** | 6.072595 | 0.002949 | 2059.542267 | 0.000000 |
| **300+0** | 6.380064 | 0.001832 | 3482.297043 | 0.000000 |
| **300+3** | 9.451563 | 0.002796 | 3380.780601 | 0.000000 |
| **600+0** | 10.775740 | 0.001617 | 6664.835927 | 0.000000 |
| **600+5** | 14.347929 | 0.014218 | 1009.113997 | 0.000000 |
| **900+10** | 21.608455 | 0.018034 | 1198.176487 | 0.000000 |
| **1800+0** | 22.579915 | 0.017313 | 1304.217586 | 0.000000 |
| **1800+20** | 24.411265 | 0.109903 | 222.116865 | 0.000000 |
| **60+0 ELO** | -0.061395 | 0.000574 | -106.888058 | 0.000000 |
| **120+1 ELO** | -0.054044 | 0.001159 | -46.611652 | 0.000000 |
| **180+0 ELO** | -0.087000 | 0.000565 | -153.973781 | 0.000000 |
| **180+2 ELO** | -0.033159 | 0.000884 | -37.509793 | 0.000000 |
| **300+0 ELO** | -0.139081 | 0.000546 | -254.580913 | 0.000000 |
| **300+3 ELO** | -0.031694 | 0.000912 | -34.763540 | 0.000000 |
| **600+0 ELO** | -0.139464 | 0.000546 | -255.194990 | 0.000000 |
| **600+5 ELO** | 0.163983 | 0.004932 | 33.250805 | 0.000000 |
| **900+10 ELO** | 0.284079 | 0.006916 | 41.075081 | 0.000000 |
| **1800+0 ELO** | 0.715882 | 0.007919 | 90.395121 | 0.000000 |
| **1800+20 ELO** | -5.323413 | 0.102739 | -51.815062 | 0.000000 |
| **60+0 EΔUC** | 1.119211 | 0.013597 | 82.314449 | 0.000000 |
| **120+1 EΔUC** | 4.255405 | 0.022367 | 190.254484 | 0.000000 |
| **180+0 EΔUC** | 4.109497 | 0.014166 | 290.092680 | 0.000000 |
| **180+2 EΔUC** | 6.780749 | 0.017657 | 384.026128 | 0.000000 |
| **300+0 EΔUC** | 6.954207 | 0.010875 | 639.446349 | 0.000000 |
| **300+3 EΔUC** | 10.518386 | 0.016320 | 644.509550 | 0.000000 |
| **600+0 EΔUC** | 11.950344 | 0.009661 | 1236.933656 | 0.000000 |
| **600+5 EΔUC** | 16.230136 | 0.085497 | 189.833007 | 0.000000 |
| **900+10 EΔUC** | 23.658403 | 0.108956 | 217.137475 | 0.000000 |
| **1800+0 EΔUC** | 22.725715 | 0.105945 | 214.505566 | 0.000000 |
| **1800+20 EΔUC** | 74.483945 | 0.768751 | 96.889534 | 0.000000 |
| **60+0 ELO & EΔUC** | 0.027654 | 0.003647 | 7.582058 | 0.000000 |
| **120+1 ELO & EΔUC** | 0.146055 | 0.006929 | 21.079756 | 0.000000 |
| **180+0 ELO & EΔUC** | 0.151163 | 0.003381 | 44.714571 | 0.000000 |
| **180+2 ELO & EΔUC** | 0.312359 | 0.005256 | 59.424115 | 0.000000 |
| **300+0 ELO & EΔUC** | 0.259564 | 0.003240 | 80.110347 | 0.000000 |
| **300+3 ELO & EΔUC** | 0.317424 | 0.005416 | 58.603818 | 0.000000 |
| **600+0 ELO & EΔUC** | 0.494467 | 0.003237 | 152.759776 | 0.000000 |
| **600+5 ELO & EΔUC** | 1.256484 | 0.028650 | 43.855961 | 0.000000 |
| **900+10 ELO & EΔUC** | 2.681459 | 0.040903 | 65.556806 | 0.000000 |
| **1800+0 ELO & EΔUC** | -0.136469 | 0.047315 | -2.884284 | 0.003923 |
| **1800+20 ELO & EΔUC** | -15.682593 | 0.709581 | -22.101203 | 0.000000 |


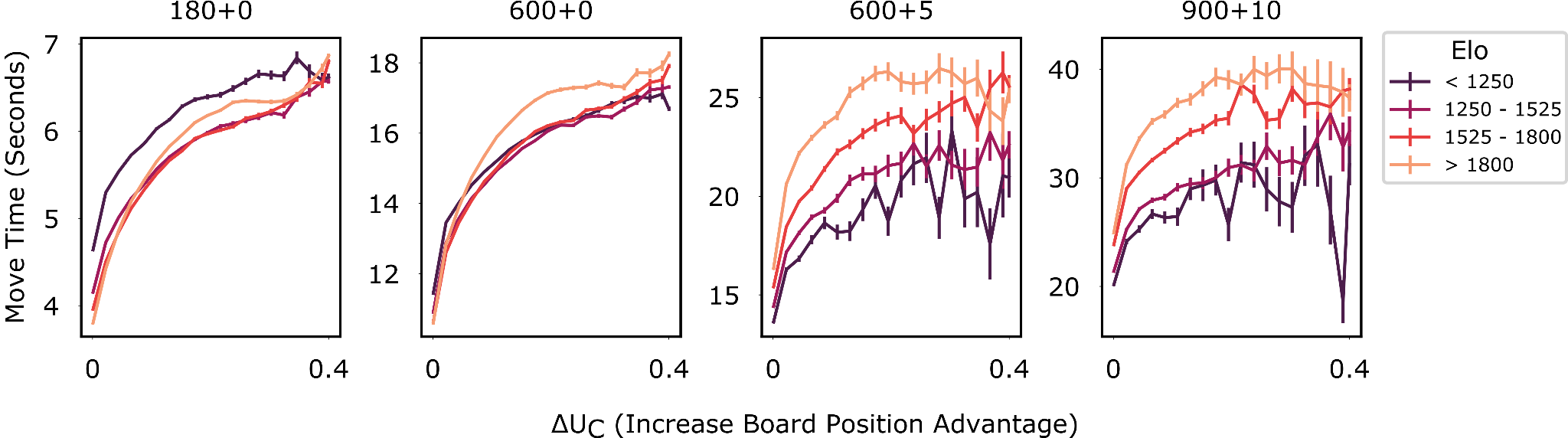


***Supplementary Figure 1****: Mean move times as a function of benefit of computation and Elo quartile for four example time-control settings.*

**Interrogating the validity and necessity of assumptions underlying the benefit of computation**

The benefit of executing a computation, $\Delta U_{C}$, is defined as the difference in true utility ($U_{C}$, board position advantage) between the optimal move that could be arrived at through computation, $m_{c}$, and a move that one could arrive at using little or less computation, $m_{-c}$:

$\Delta U_{C}=U_{c}\left( m_{c} \right)-U_{C}(m_{-c})$

Our use of Stockfish chess engine to define $\Delta U_{C}$ makes use of two implicit assumptions. The first is that Stockfish with computation (which we took to be depth-15) provides both the optimal move, $m_{c}$, and the true utility over moves, $U_{c}$. The second is that Stockfish at depth-1 (which simply evaluates a neural network static evaluator to a resultant board state) provides an appropriate model of move selection without computation, $m_{-c}$.

We first examine the validity of these assumptions by seeing whether they describe how players moves change as they spend more time due to either increasing benefit of computation or time-contro.. Subsequently, we investigate the necessity of these assumptions for our results. To do this, we by first redefining $U_{c}$ and $m_{c}$ with greater computation than depth-15 search, which increases the validity of the first assumption. We also use alternate models for selection of $m_{-c}$ which tests the robustness of our results to violations of the second assumption. We demonstrate that our key findings – namely the relationship between move times and benefit of computation, $\Delta U_{C}$, and the relationship between this effect itself and player Elo – are robust to different approaches to move selection and defining $\Delta U_{C}$.

**1. Testing the validity of assumptions**

Our definition of the benefit of computation implies that when individuals spend more time thinking, their moves should increasingly reflect Stockfish’s selections at depth-15 compared to depth-1. Here, we investigate whether players' increased thinking time shifts the predictive balance from depth 1 to depth 15.

We note that to properly test this, it is not sufficient to simply analyze how the accuracy of depth-1 versus depth-15 Stockfish in predicting players’ moves changes with varying move times. This is because both prediction accuracy and move time can be influenced by factors unrelated to computation benefit, such as move difficulty (e.g., players may spend more time on more challenging positions, which could also lower prediction accuracy). To control for such latent factors, we examine changes in Stockfish's depth-1 and depth-15 prediction accuracy as a function of move time, using variance induced by two factors shown in the main manuscript to affect move time: the benefit of computation, and time-control settings. Thus, we bin moves based on the time-control setting (11 total) and the benefit of computation (11 bins ranging from 0 to 0.2). For each bin, we calculate the mean response time and the mean accuracy of Stockfish at depth-1 and depth-15 in predicting the moves actually played by the human players.

Supplementary Fig. 2 presents the mean prediction accuracy of Stockfish at depth-1 and depth-15 as the benefit of computation varies, separately for each time-control setting. Within each time-control setting, at the lowest values of $\Delta U_{C}$, Stockfish at depth-1 predicts players' moves better than Stockfish at depth-15. As $\Delta U_{C}$ increases (moving rightward in each panel), players take more time to decide, and the predictive accuracy of depth-15 relative to depth-1 increases, thus confirming the expected effect of increasing benefit of computation and move time.


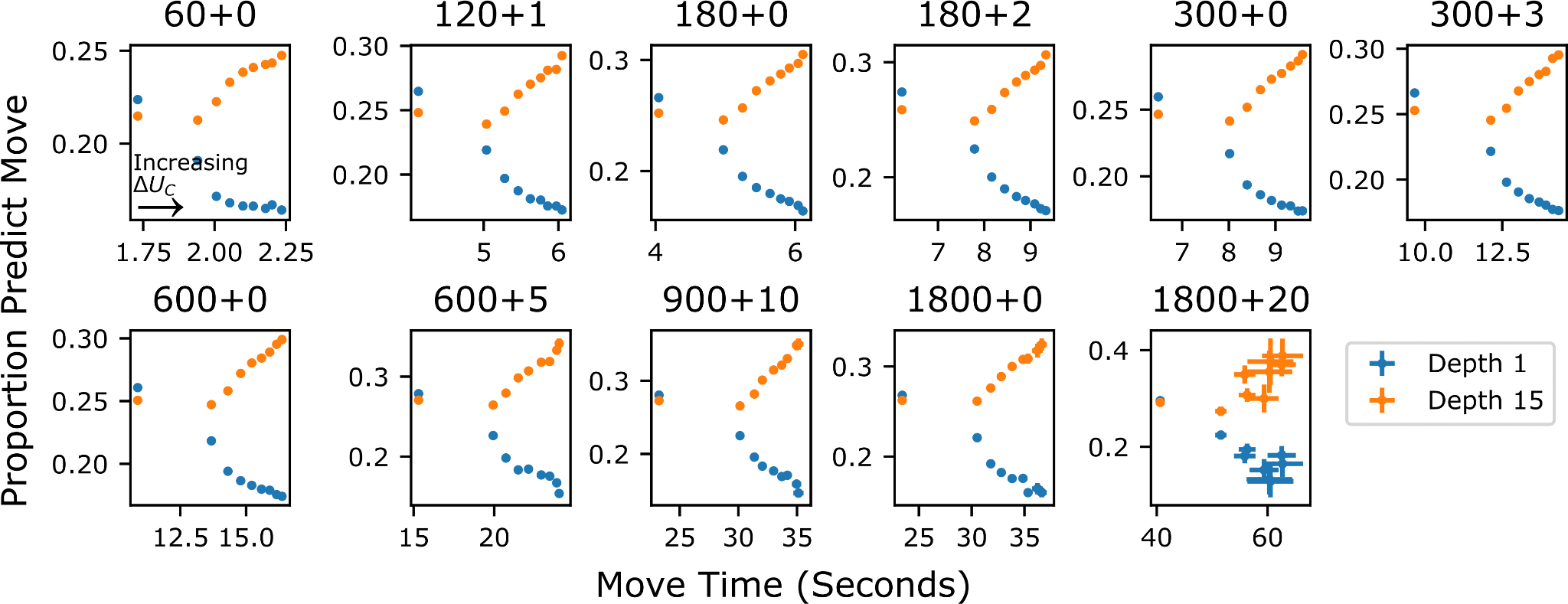


**Supplementary Figure 2.** Move prediction accuracy of Stockfish at Depth-1 and Depth-15 as the benefit of computation is varied. Each panel shows a different time-control setting. As $\Delta U_{C}$ increases, players spend more time and the their moves become better predicted by Depth-15 than Depth-1 Stockfish. Error bars denote standard error of the mean (horizontal) and standard error of proportion (vertical).

Supplementary Fig. 3 presents the same data, but varies time-control setting within each panel. As time-control setting varies, move times increase and the prediction accuracy o Stockfish atf depth-15 relative to depth-1 increases. Together, these findings confirm the expected relationship between response time and move selection, whether response time varies due to having additional time or the choice to spend more time based on a higher benefit of computation. This provides further validation for our definition of the benefit of computation.


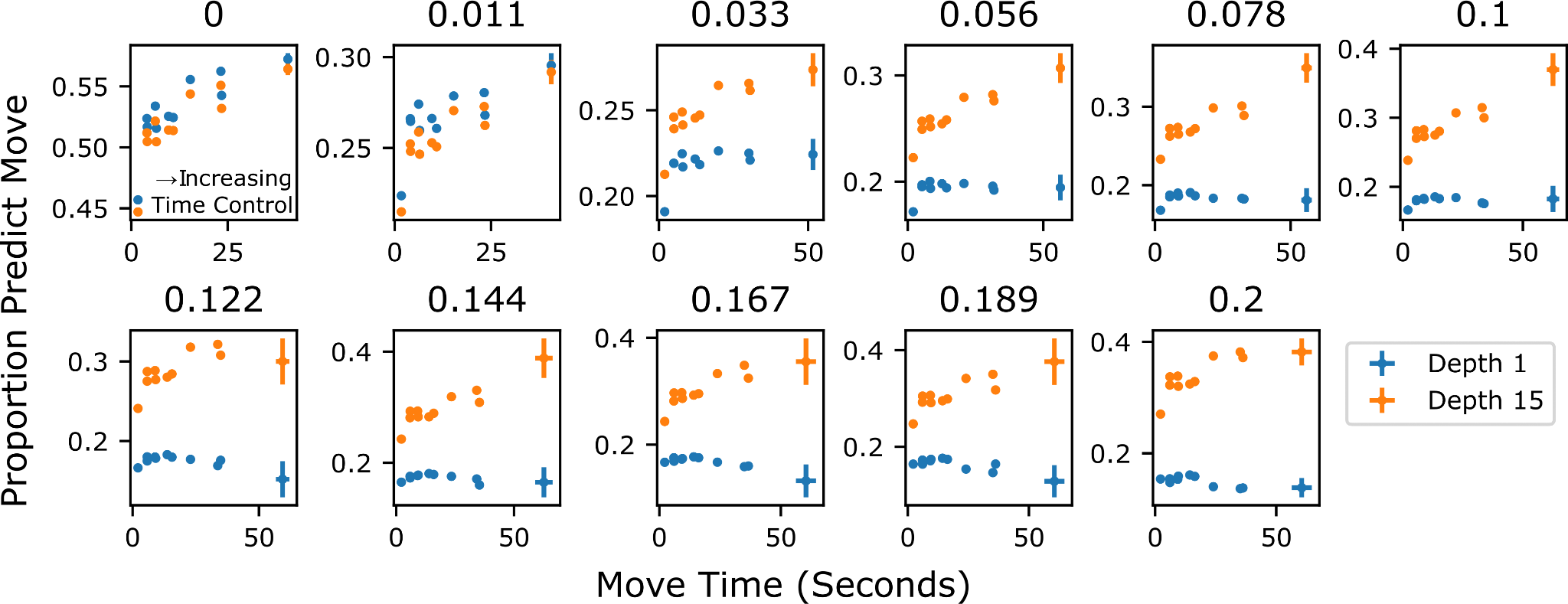


**Supplementary Figure 3.** Move prediction accuracy of Stockfish at Depth-1 and Depth-15 as the time-control setting is varied. Each panel shows a benefit of computation, $\Delta U_{C}$. As time-control setting increases, players spend more time and the their moves become better predicted by Depth-15 than Depth-1 Stockfish. Error bars denote standard error of the mean (horizontal) and standard error of proportion (vertical).

**2. Alternate models of effects of computation- defining** $\Delta U_{C}$ **with different** $U_{C}$ **and** $m_{c}$

Under our idealized model of the effects of computation, we posit that computation can in principle provide both the optimal move in a situation, $m_{c}$, as well as the true utility of moves under consideration, $U_{C}$.

In our main analysis, we approximate this “true” utility function, $U_{C}(m_{i})$ , as stockfish’s depth-15 evaluation of $m_{i}$. In practice, depth-15 was selected based on norms for high stockfish evaluation set in previous studies [(McIlroy-Young et al., 2020)](https://paperpile.com/c/UzogcO/OYQ2), and based on practical considerations around time taken to perform evaluations. However, depth-15 stockfish sometimes does not define the ‘true’ utility function, nor identify the optimal move.

In this section, we thus test whether such errors due to this approximation could affect our results, by examining whether our results change if we make this approximation closer to ground truth. We do this by defining $U_{C}$ and $m_{c}$ under a range of increasing depths (reflecting more accurate estimates of move utility), and examining whether our results change if $U_{C}$ more reflects the ‘true’ utility function. These analysis were performed on 111k games from the 600+0 time-control setting.

Supplementary Table 6 provides the Spearman correlation between $\Delta U_{C}$, defined with $U_{C}$ as depth-15 stockfish, and $U_{C}$ defined using some higher depth (16 through 20). As seen, these different versions of $\Delta U_{C}$ are highly correlated, demonstrating that depth-15 stockfish provides a good approximation of the ‘true’ move utility.

**Supplementary Table 6**: Spearman correlation between $\Delta U_{C}$, defined with $U_{C}$ as depth-15 stockfish, and $U_{C}$ as a higher depth (16 through 20).

|  | **Depth 16** | **Depth 17** | **Depth 18** | **Depth 19** | **Depth 20** |
| --- | --- | --- | --- | --- | --- |
| **Corr. w Depth 15** | 0.874174 | 0.847153 | 0.830303 | 0.819819 | 0.811167 |

Supplementary Figure 2 presents the results of our two key analysis - relationship between move times and benefit of computation, $\Delta U_{C}$ (top), and the relationship between this effect and player Elo (bottom) using each of these versions of $\Delta U_{C}$. As observed, these effects are strongly observed for each variation of $\Delta U_{C}$ (Effect of $\Delta U_{C}$ on move time: all estimates > 13.9, all P < 1e50; Interaction with Elo: all estimates > .0044, all P < 1e50).


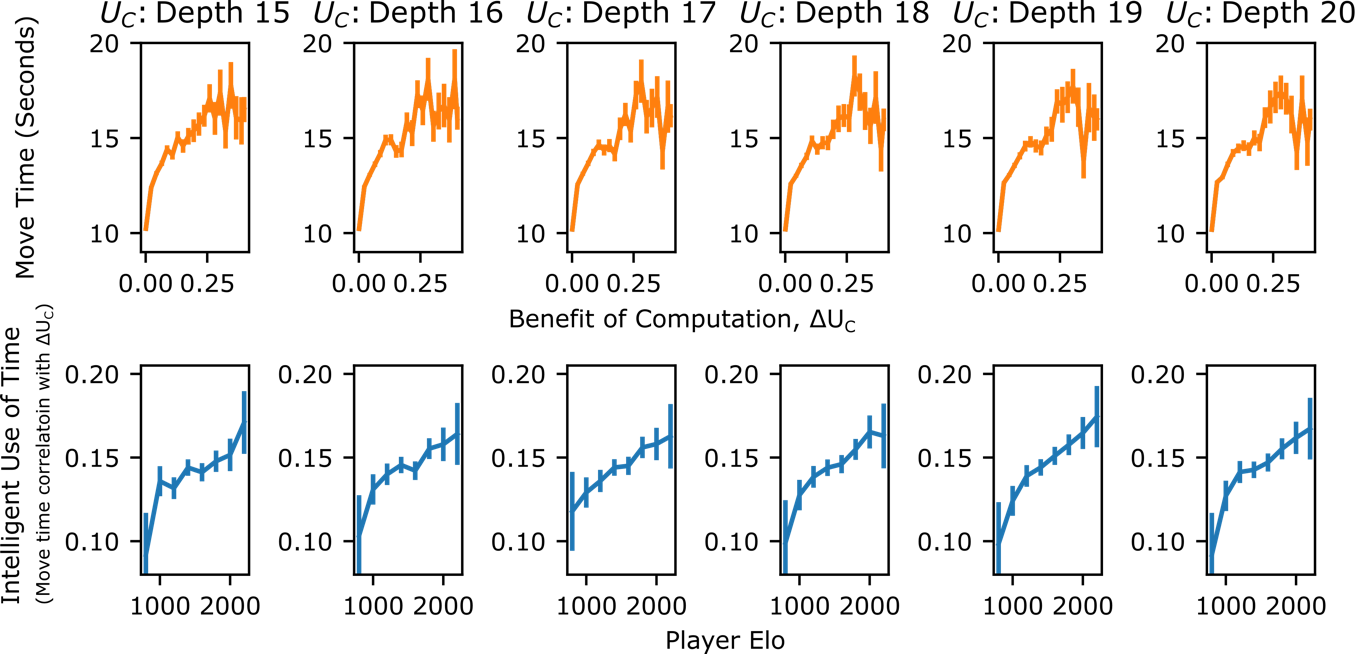


***Supplementary Figure 4****. Key relationships observed for different versions of* $\Delta U_{C}$*, where* $U_{C}$ *and* $m_{C}$ *are defined with increasing depth (horizontal panel). Top: Relationship between move times and benefit of computation,* $\Delta U_{C}$*. Bottom: Relationship between effect of* $\Delta U_{C}$ *on move time, and player Elo.*

**3. Alternate models of move selection with no computation - defining** $\Delta U_{C}$ **with different** $m_{-c}$

The benefit of computation is also dependent on the move individuals would select without performing computation, $m_{-c}$. In the main text, we define $m_{-c}$ as the move selected by stockfish given a depth-1 search. However, we acknowledge that this reflects an approximation to how individuals in practice select moves without computation. In this section, we examine the robustness of our results to challenging this approximation, by repeating key analysis under alternate ways of defining $m_{-c}$.

Overall, we hope to demonstrate that the benefit of computation reflects an abstract notion of the benefits of planning deeper in heuristic tree search. Board positions with high $\Delta U_{C}$ reflect situations where the benefits of performing the optimal move are much greater than the move one would perform with *less* computation. Importantly, identifying such positions does not depend on having a perfect model of human move selection without computation. Rather such positions can be identified through a variety of approach to find moves that one would select with limited tree search.

We first demonstrate this by recomputing $\Delta U_{C}$, defining $m_{-c}$ using Stockfish evaluation depths greater than depth-1. These different $\Delta U_{C}$, defined with $m_{c}$ at greater depths, demonstrate relationships with move time, and these relationships increase with increasing player Elo (Supplementary Fig. 3; (Effect of $\surd\Delta U_{C}$ on move time: all estimates > 11.3, all P < 1e50; Interaction with Elo: all estimates > .0040, all P < 1e50).


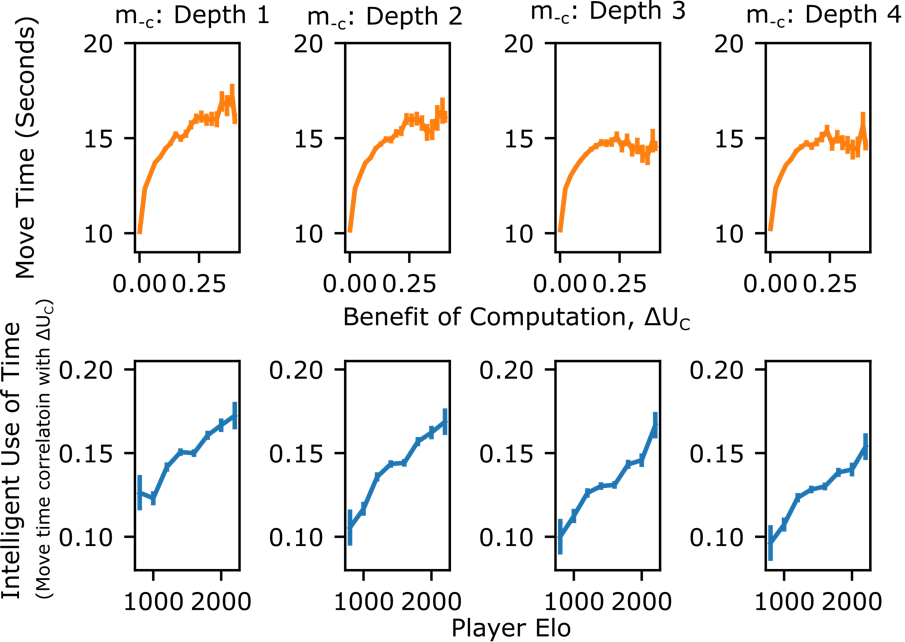


***Supplementary Figure 5****. Key relationships observed for different versions of* $\Delta U_{C}$*, where* $m_{-c}$ *is selected with different, increasing depths (horizontal panel). Top: Relationship between move times and benefit of computation,* $\Delta U_{C}$*. Bottom: Relationship between effect of* $\Delta U_{C}$ *on move time, and player Elo.*

We next investigated whether our results would be robust to using a different model of how moves are selected without computation, and in particular investigated selection of $m_{-c}$with different engines. We first recomputed $\Delta U_{C}$, using Stockfish version 8 to select moves without computation, $m_{-c}$. Note that we still use Stockfish-14 to select the optimal move and to evaluate moves under true utilities. Stockfish version 8 is substantially different than version 14 – notably in that it does not use a neural network as a static evaluation function at all – but rather forms a static evaluation function using hand-designed human-defined features. We found that $\Delta U_{C}$ for Stockfish 8 was positively related to move times, and this relationship increased with player Elo (Supplementary Fig. 4; Effect of $\surd\Delta U_{C}$ on move time estimate = 9.66, P < 1e50; Interaction with Elo: Estimate = .0017, P < 1e50).

**
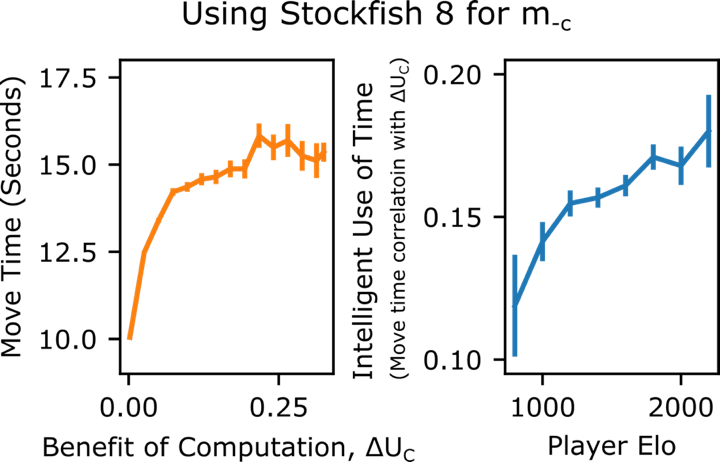
**

***Supplementary Figure 6.*** *Defining* $\Delta U_{C}$ *where* $m_{c}$ *is identified using Stockfish-8. Left: Relationship between move times and benefit of computation,* $\Delta U_{C}$*. Right: Relationship between effect of* $\Delta U_{C}$ *on move time, and player Elo. Analysis performed on 18k games from 600+0 time-control setting.*

We next tested, a model which selected $m_{-c}$ using the Leela-chess zero policy network (<https://lczero.org/>). Leela-chess zero is an open-source implementation of Alpha-chess zero [(Silver et al., 2018)](https://paperpile.com/c/UzogcO/c6qI). Notably, although Leela-chess zero also works by combining neural networks with tree search, the details of the neural network and how it is trained are quite different from Stockfish. Here, we used the Leela-chess zero network to define the move that would be selected without computation but use depth-15 Stockfish preferred move for $m_{c}$, the optimal move discovered with computation, and $U_{c}$, the true utility of each move. $\Delta U_{C}$ defined with Leela-chess zero was still related to move times, and this relationship increased with player Elo (Supplementary Fig. 5; (Effect of $\surd\Delta U_{C}$ on move time Estimate = 14.82, P < 1e50; Interaction with Elo: Estimate = .0073, P < 1e50).


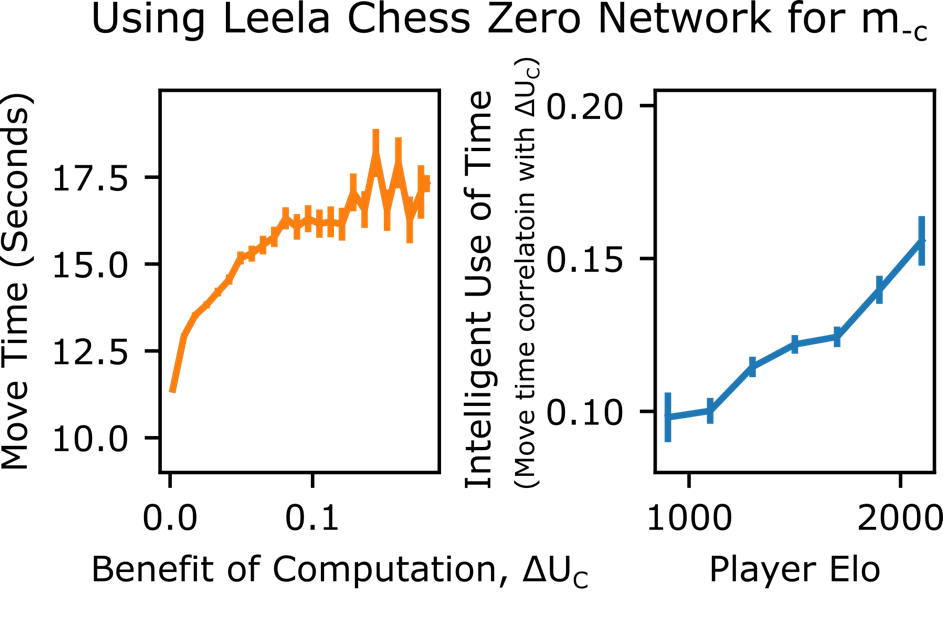


***Supplementary Figure 7.*** *Defining* $\Delta U_{C}$ *where* $m_{c}$ *is identified using Leela-chess zero policy network. Left: Relationship between move times and benefit of computation,* $\Delta U_{C}$*. Right: Relationship between effect of* $\Delta U_{C}$ *on move time, and player Elo.*

We note that the benefit of computation, $\Delta U_{C}$, was smaller when defined with Leela-chess zero than with Stockfish, and this is reflected in the range of values over which we plot the relationship (note that plot x axis shows up to 99 percentile of $\Delta U_{C}$). This occurs because the Leela-chess zero network, with no additional search, is a substantially stronger player than the Stockfish neural network, with no search, thus making the benefit of deep search relatively smaller [(Meloni, 2021)](https://paperpile.com/c/UzogcO/nYpl).

**3. Controlling for relationships between player Elo and** $\Delta U_{C}$**.**

A potential alternative explanation for our results that player Elo affects the strength of relationship between move-time and $\Delta U_{C}$ would be if instead $\Delta U_{C}$ was different for different players, in a way that related to their Elo. A more accurate $\Delta U_{C}$ would result in a stronger move-time to $\Delta U_{C}$ relationship independant of a player’s ability to recognize beneficial computational situations. Because, under our model, we define $U_{C}$ to be the ‘true’ move utility and $m_{c}$ to be the true best move, these components of the benefit of computation are not affected by player Elo (e.g. the ‘true’ utility of a move and the true best move are not related to the player making the move). In contrast, $m_{-c}$ is meant to reflect the move a player would select without applying computation. It is possible that, when $m_{-c}$ is defined using Stockfish or Leela-chess zero, that this assumption is more true for better players with higher Elo. This could lead to $\Delta U_{C}$ effectively being more accurately defined for higher rated players which could in principle underlie it’s greater relationship to move times in players with higher Elo.

To address this, we sought to define a version of $\Delta U_{C}$ where $m_{-c}$ would be more likely to correspond to moves made by players with lower compared to higher elo. For this we utilized the Maia neural network [(McIlroy-Young et al., 2020)](https://paperpile.com/c/UzogcO/OYQ2). Maia is a neural network, which utilizes the Leela-chess-zero architecture, but is trained to predict moves made by players at a certain Elo rating. The weakest Maia agent (Maia-1100) is trained specifically to predict the moves of players with an Elo of 1100, and predicts their moves with greater accuracy than moves made by players with higher Elo.

We thus defined $\Delta U_{C}$, defining $m_{-c}$ as the move selected by Maia-1100. We note that one limitation of this approach is that $m_{-c}$ is meant to reflect moves selected without computation, however Maia-1100 is trained on all moves made by players with Elo 1100. Nevertheless, we suggest that, Maia-1100 is likely a better predictor of low computation moves of weaker players than stronger players, thus enabling its use for testing the validity of this potential alternative explanation to the relationship between Elo and effectiveness of time spent.

The benefit of computation, $\Delta U_{C}$, defined using Maia-1100 was still positively related to move times, and this effect still positively increased with player Elo (Supplementary Fig. 6; Effect of $\Delta U_{C}$ on move time = 12.43, P < 1e50; Interaction with Elo: estimates = .0026, P < 1e50). This suggests that the relationship of effect of $\Delta U_{C}$ on move times and player Elo is not due $\Delta U_{C}$ being more aligned with moves made by stronger players.


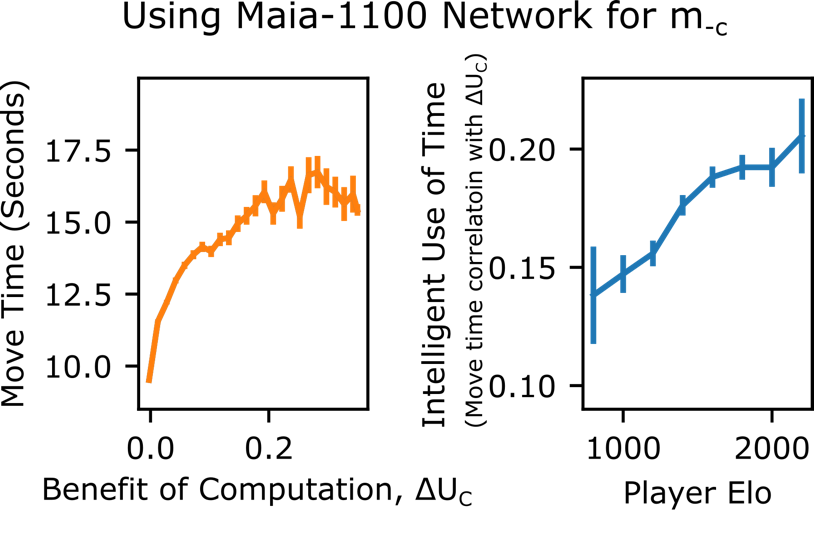


***Supplementary Figure 8.*** *Defining* $\Delta U_{C}$ *where* $m_{-c}$ *is identified using Maia-1100. Left: Relationship between move times and benefit of computation,* $\Delta U_{C}$*. Right: Relationship between effect of* $\Delta U_{C}$ *on move time, and player Elo.*

**Additional figures for cost of time analysis**

**
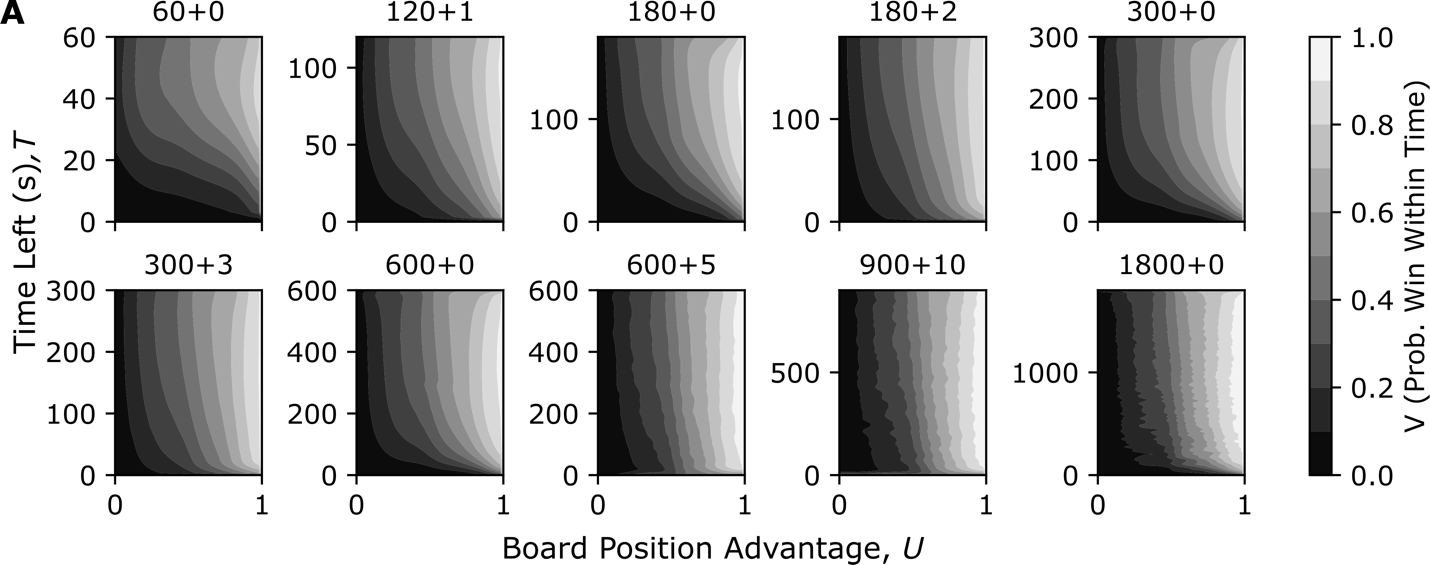
**

***Supplementary Figure 9****.* $V_{tc}$ *for each time-control setting.* $V_{tc}\left( T,U \right)$ *is the empirical probability of winning a match following for time-condition tc, following a game state with time-left T and board position advantage U. Note that for all time-control settings, .as T approaches 0,* $V_{tc}\left( T,U \right)$ *shifts gradually from being more influenced by board position advantage to time remaining. This in turn causes the cost of time to increase nonlinearly and depend on time remaining.*


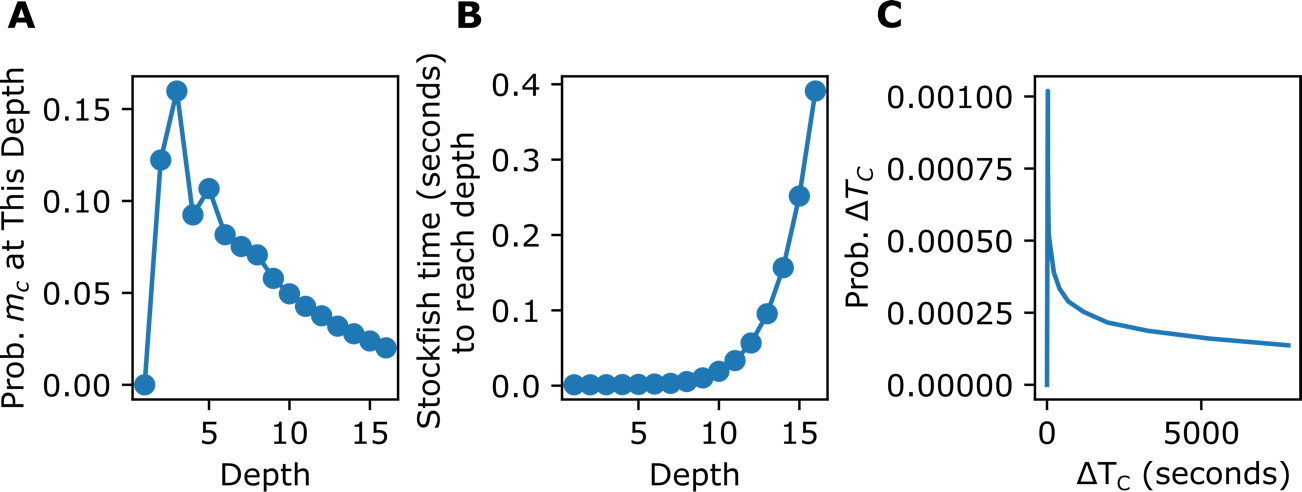


***Supplementary Figure 10: Computation of*** $P\left( \Delta T_{C} \right)$***.*** *We computed a probability distribution over the time that stockfish takes to arrive at the move,* $m_{c}$*, which maximizes a depth-15 value function,* $U_{c}$*, and converted this to a* $P\left( \Delta T_{C} \right)$ *by multiplying each* $\Delta T_{C}$ *by a scaling parameter, set to 20000. A. We first measured the probability over depths that Stockfish arrived at* $m_{c}$ *at that depth. B. This was combined with the average time taken by Stockfish to reach a given depth in seconds. C. We arrived at* $P\left( \Delta T_{C} \right)$ *by multiplying each Stockfish time by a scaling parameter and interpolating.*


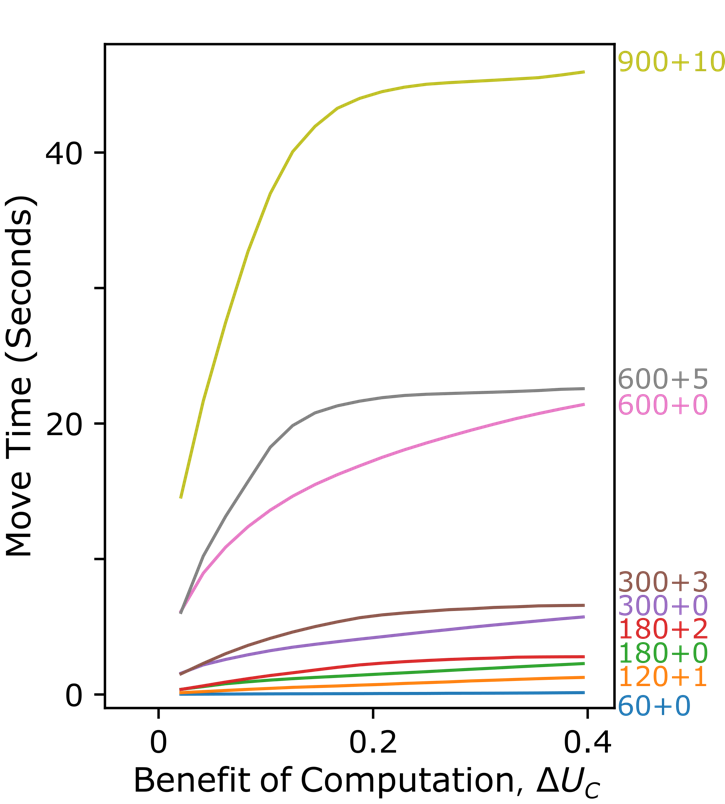


**Supplementary Figure 11. *Mean move times implied by empirically measured cost structure.*** *Mean move times for optimal policy over distribution of computational offers estimated empirically. Equivalent to Fig. 7D, but with all time-control settings shown through 900+10 (for visualization purposes. Empirically measured cost structure can underlie concave increase in move times with benefit of computation, and dependence of move times on both total clock time and increment. Subset of time-control settings are displayed to enable visualization of key qualitative effects.*


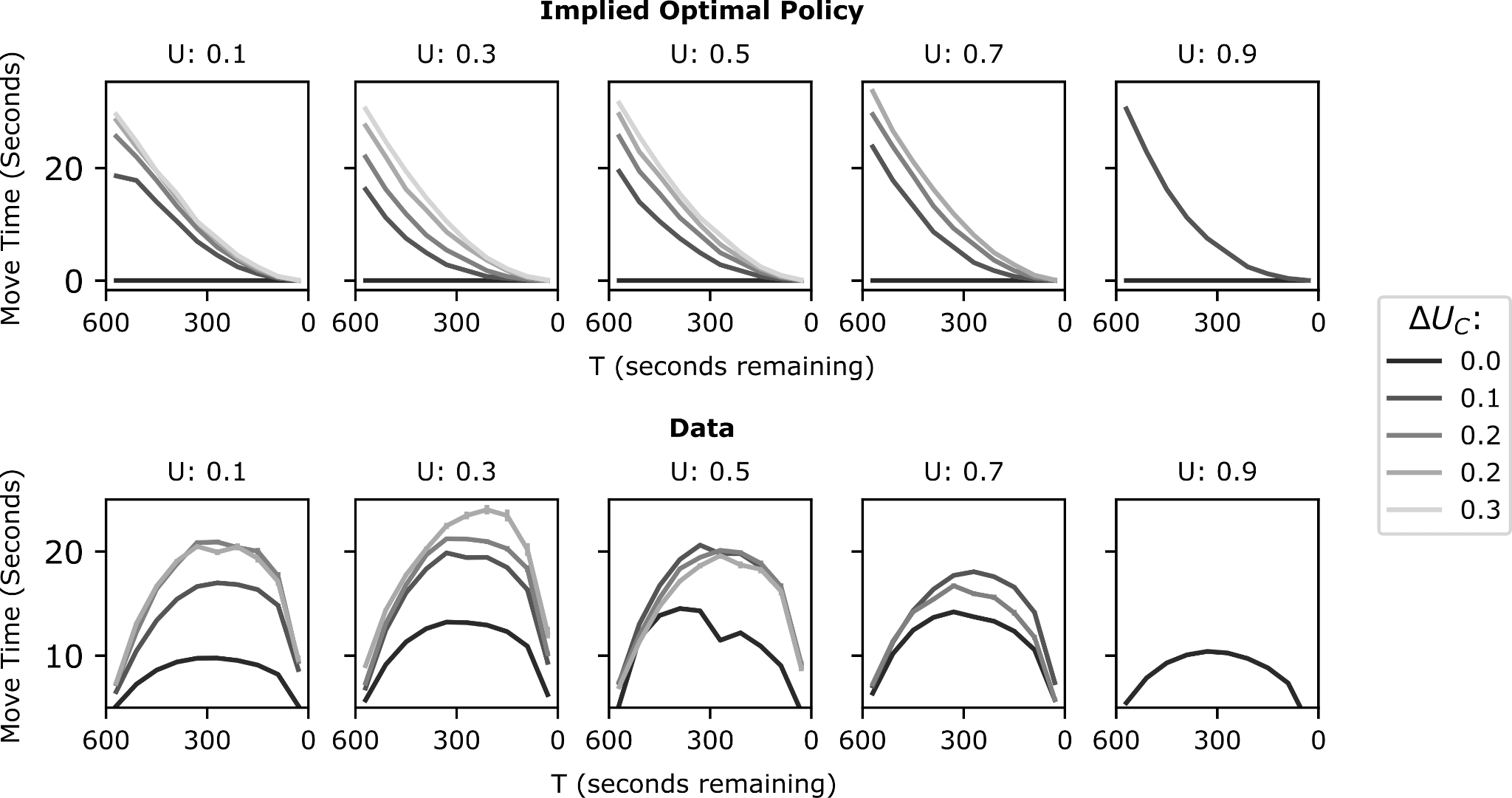


***Supplementary Figure 12: Discrepancies between effects of within game changes of time remaining (T) on implied optimal policy predicted and measured move times. A****. Implied optimal policy (from win-rates over time-left and board position advantage) predicted mean move times from the 600+0 time-control setting as a function of* $U$*,* $T$ *and* $\Delta U_{C}$*. The implied optimal policy predicts that mean move times should decrease as T approaches 0.* ***B.*** *Measured mean move times from the 600+0 time-control as a function of* $U$*,* $T$ *and* $\Delta U_{C}$*. Observed move times increase and then decrease as T approaches 0. This discrepancy may reflect the use of clock time as a feature with which to estimate* $\Delta U_{C}$*, as moves at the start of games tend to have low* $\Delta U_{C}$*.*


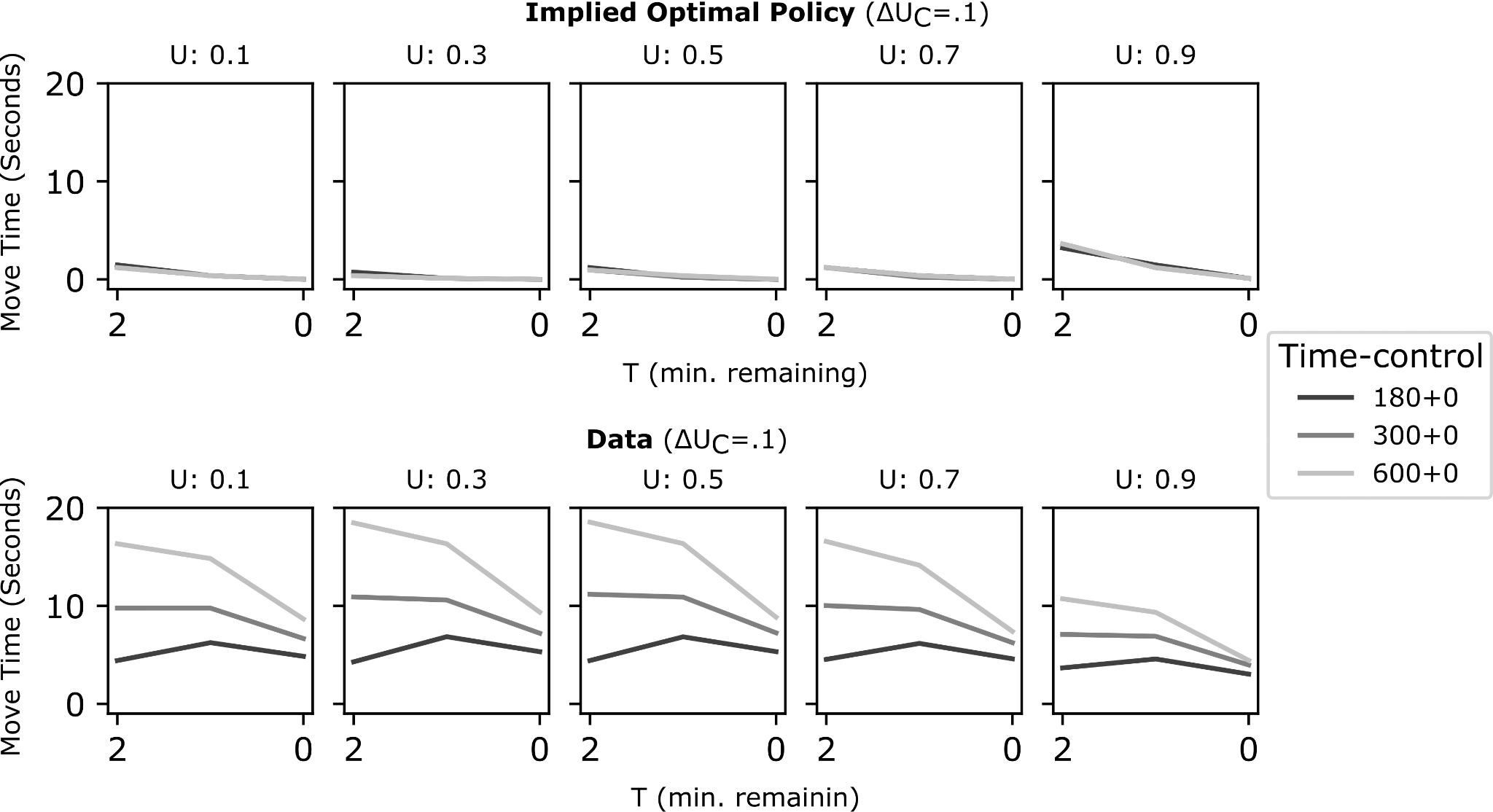


***Supplementary Figure 13: Discrepancies between effects of time-control setting on move-times between implied optimal policy predicted and measured move times****.* ***A****. Implied optimal policy (from win-rates over time-left and board position advantage) predicted mean move times from one of three time-control settings as a function of* $U$ *and* $T$*, taken at* $\Delta U_{C}=.1$*. The optimal policy does not predict an effect of time-control setting on mean move-times after controlling for* $U$*, T and* $\Delta U_{C}$*.* ***B.*** *In contrast, measured mean move-times display large effects of time-control setting even after controlling for U, T and* $\Delta U_{C}$*. This discrepancy may suggest that individuals approximate this optimal policy by pre-computing the average move time for a given* $\Delta U_{C}$ *separately for each time-condition and reusing this without re-computing the optimal move time prior to each move.*
